# Development of programmable RNA imaging with RNA-guided GFP via click chemistry

**DOI:** 10.1093/nar/gkaf1147

**Published:** 2025-11-08

**Authors:** Jun Nakamura, Miyako Shiraishi, Junpei Yamamoto, Keiichiro Suzuki

**Affiliations:** Graduate School of Frontier Bioscience, The University of Osaka, 1-3 Yamadaoka, Suita, Osaka 565-0871, Japan; Graduate School of Engineering Science, The University of Osaka, 1-3 Machikaneyama, Toyonaka, Osaka 560-8531, Japan; Graduate School of Pharmaceutical Sciences, Kyushu University, 3-1-1 Maidashi, Higashi-ku, Fukuoka 812-8582, Japan; Graduate School of Engineering Science, The University of Osaka, 1-3 Machikaneyama, Toyonaka, Osaka 560-8531, Japan; Graduate School of Frontier Bioscience, The University of Osaka, 1-3 Yamadaoka, Suita, Osaka 565-0871, Japan; Graduate School of Engineering Science, The University of Osaka, 1-3 Machikaneyama, Toyonaka, Osaka 560-8531, Japan; Institute for Advanced Co-Creation Studies, The University of Osaka, 1-3 Machikaneyama, Toyonaka, Osaka 560-8531, Japan

## Abstract

The CRISPR-Cas system revolutionized molecular biology by guiding Cas proteins to target nucleic acid sequences using customizable guide RNAs, offering unparalleled precision and versatility. Inspired by this innovation, we developed RNA-guided green fluorescent protein (RGG), a simple and programmable platform for targeting nucleic acid. Using a streamlined click chemistry approach, known for its high efficiency and specificity, we conjugated dibenzocyclooctyne (DBCO)-modified guide nucleic acids, designed to complement target sequences, with azide-exposed proteins to construct RGG. Systematic optimization identified 30-nt RNA with 3′-DBCO modifications as the most effective configuration for RGG, enabling precise visualization of nuclear-localized RNAs, including *NEAT1* and Satellite III RNA, in living cells. This establishes RGG as a customizable and efficient system for RNA imaging and molecular analysis, underscoring the potential of direct conjugation between guide nucleic acids and proteins to enable precise nucleic acid recognition and dynamic molecular modification in living cells.

## Graphical Abstract

**Graphical Abstract:**
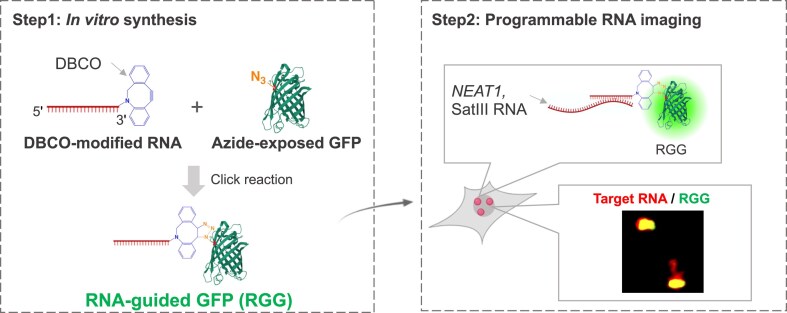

## Introduction

The advent of the CRISPR-Cas system, especially CRISPR-Cas9, revolutionized genome editing by providing a programmable tool that uses guide RNA (gRNA) to direct Cas proteins to specific target sequences, where they execute precise cleavage [1, 2]. Among these, CRISPR-Cas13 stands out as a unique RNA-targeting system that use its RNase activity to cleave single-stranded molecules in a sequence-specific manner [3]. Unlike DNA-targeting Cas9, Cas13 recognizes RNA targets without requiring a protospacer adjacent motif [4]. While some Cas13 orthologs require a protospacer flanking sequence, many widely used variants for RNA imaging—such as catalytically inactive dLwaCas13a, dPguCas13b, and dPspCas13b—do not, thereby expanding broadening their applicability in RNA biology [5, 6]. Cas13 variants have been further engineered to expand functionality, including RNA editing, base modification and live-cell RNA imaging [3, 6]. These innovations enable CRISPR-Cas systems to surpass earlier tools such as zinc-finger nucleases and transcription activator-like effector nucleases, which rely on intricate protein engineering to recognize specific DNA sequences [7, 8]. In contrast, CRISPR-Cas relies on RNA-guided recognition, offering a straightforward, cost-effective, and highly adaptable design, has become an invaluable tool in molecular biology and biotechnology. Beyond genome editing, CRISPR-Cas technologies have been adapted for transcriptional regulation, RNA targeting, and live-cell imaging, showcasing their versatility as a platform for molecular manipulation and discovery [3, 6, 9, 10].

Building on the transformative success of CRISPR-Cas systems, researchers have developed innovative RNA-guided protein technologies that offer similar programmability and versatility. One notable example is the CRISPR-Cas-inspired RNA targeting system (CIRTS), which combines gRNAs linked to a hairpin RNA sequence with effector proteins fused to hairpin RNA-binding domains [11]. This modular design enables a wide range of functionalities, including RNA cleavage, editing and translation regulation, simply by altering the effector protein. As a genetically encoded platform, CIRTS represents a significant step forward in RNA manipulation tools. Another innovative strategy leverages chemically modifying gRNAs with benzylguanine, enabling conjugation with the SNAP-tag, a high-affinity binding protein [12, 13]. In this system, the modified gRNA is introduced into cells expressing an RNA editing protein, facilitating conjugation between the gRNA and the editing protein. This system achieves precise RNA editing, including A-to-I or C-to-U modifications, and allows fine control over gRNA dosage for enhanced precision and efficiency. These advancements in RNA-guided tools, encompassing RNA-effector proteins complexes and RNA–protein conjugates, hold immense potential for precise RNA targeting.

Click reactions are widely recognized for their robustness, high chemical yields and exceptional selectivity, functioning efficiently under diverse aqueous and physiological conditions with minimal by-product formation [14]. Among these, strain-promoted azide-alkyne cycloaddition (SPAAC) stands out for its unique advantages [15]. Unlike copper-catalyzed reactions, which can introduce toxicity, SPAAC employs azide, and cyclooctyne functional groups, ensuring high biocompatibility. The functional groups involved in SPAAC are considerably smaller compared to bulky protein tags, minimizing interference with biological processes. Furthermore, SPAAC’s remarkable specificity prevents unintended interactions with cellular components, making it an ideal choice for precise chemical tagging. These properties have enabled SPAAC to find broad applications across various fields [16, 17]. One notable application involves metabolic engineering, where cells are expose to azide-containing compounds, resulting in azide-functionalized proteins on the cell surface [17]. Subsequently, introducing a dibenzocyclooctyne (DBCO, cyclooctyne derivative)-conjugated fluorescent dye into the culture medium facilitates the selective fluorescent labelling of living cell surfaces via SPAAC. This versatility highlights SPAAC’s utility in diverse research areas, including cellular imaging and chemical biology, firmly establishing it as a highly valuable tool in modern scientific studies.

Imaging intracellular RNA is crucial for uncovering RNA function, as it reveals the spatial distribution and dynamics of RNA within living cells. Traditional techniques like fluorescence *in situ* hybridization (FISH) provide high sensitivity and quantitative data but require cell fixation, restricting their use to static observations [18]. For live-cell RNA imaging, the MS2-MCP system is a widely used method. This approach employs the bacteriophage MS2 coat protein (MCP), which binds specifically to MS2 stem-loop RNA sequences [19]. RNA visualization is achieved by fusing MCP to a fluorescent protein and incorporating multiple copies of the MS2 stem-loop sequence into the target RNA. Thanks to its genetically encoded nature, the MS2-MCP system is cost-effective, simple to design and widely applied in studying RNA dynamics in yeast and mammalian cells [20, 21]. However, the requirement to artificially integrate multiple MS2 sequences into the target RNA can disrupt its native function, structure, or localization, posing significant limitations [22, 23]. Considering these limitations, CRISPR-based platforms have emerged as powerful tools for RNA imaging. The CRISPR-Cas13 system, for example, enables the fluorescent labelling of endogenous transcripts without modifying their sequence. This system uses dCas13b fused to a fluorescent protein and directed by a gRNA to specifically bind the target RNA [6], allowing dynamic tracking of native RNAs in living cells. More recently, type III CRISPR-Cas systems have been adapted for RNA visualization, expanding the RNA-targeting capabilities of CRISPR technologies. These systems exploit multicomponent RNA-guided surveillance complexes for improved signal amplification and spatial resolution in live cells [24, 25]. Despite these advances, CRISPR-based approaches rely on relatively large protein complexes and RNA–protein interactions, which may complicate multiplexing or introduce potential perturbations. Moreover, aptamer-based systems like Spinach and Mango have also been developed to visualize RNA through fluorogenic RNA–fluorophore complexes, but these require complex RNA engineering and can be sensitive to cellular conditions [26, 27].

In this study, we introduce a novel, chemically defined RNA imaging platform that offers a modular, compact, and genetically noninvasive alternative. By directly conjugating DBCO-modified gRNAs to azide-functionalized fluorescent proteins via SPAAC, we enable precise and efficient live-cell imaging of endogenous RNAs. Unlike systems that require co-expression of RNA and protein components, our approach leverages bio-orthogonal click chemistry to establish a covalent RNA–protein complex exogenously, eliminating the need for genetic manipulation of the target transcript. This strategy complements and extends current RNA imaging technologies, providing a versatile platform for studying RNA localization and dynamics in live cells.

## Materials and methods

### Plasmid construction

Several plasmids used in this study were obtained from Addgene, including pULTRA-CNF (Addgene 48215), pET28a-mH6-Cas12c1 (Addgene 120872), pmScarlet-I3_C1 (Addgene 189756), AAVS1 SA-2A-puro-pA donor (Addgene 22075), gRNA_Cloning Vector (Addgene 41824), pCAG-1BPNLS-Cas9-1BPNLS-2AGFP (Addgene 87109), eGFP L202 Reporter (Addgene 119129), pMD2.G (Addgene 12259), pMDLg/pRRE (Addgene 12251), pHAGE-IRES-puro-NLS-dPspCas13b-3xEGFP-NLS-3xFlag (referred as dCas13b, Addgene 132398), and pC0043-PspCas13b crRNA backbone (Addgene 103854).

To construct the 151TAG-GFP vector, pET28a-mH6-Cas12c1 was digested with Swa I (NEB) and EcoR I (NEB). The codon-optimized 151TAG-GFP DNA sequence was synthesized by GENEWIZ and inserted using In-Fusion HD Cloning Kit (Takara), resulting in the pET28a-151TAG-GFP plasmid (Supplementary Table S1). RNA was extracted from HeLa cells using the TRIzol RNA extraction reagent (Invitrogen), and complementary DNA (cDNA) was synthesized using the SuperScript IV First-Strand Synthesis System (Invitrogen). *NONO* and *HSF1* cDNA sequences were amplified from the HeLa cDNA pool using PrimeSTAR GXL DNA polymerase (Takara). To generate NONO-mScarlet-I3 donor and HSF1-mScarlet-I3 vectors, the mScarlet-I3 sequence was amplified from pmScarlet-I3_C1 using Q5 Hot Start High-Fidelity DNA Polymerase (NEB). For the NONO-mScarlet-I3 donor, the AAVS1 SA-2A-puro-pA donor was linearized with Sbf I (NEB) and Not I (NEB), followed by insertion of the *NONO* cDNA and mScarlet-I3 fragments using the In-Fusion HD Cloning Kit. To construct the pLenti-mScarlet-I3-HSF1, the mScarlet-I3 and *HSF1* cDNA fragments were inserted into the lentiviral backbone plasmid (eGFP L202 Reporter) which was previously digested with Age I (NEB) and Kpn I (NEB). For NONO gRNA expression vector, the gRNA_Cloning Vector was digested with Afl II (NEB), and the *NONO*-targeting gRNA spacer sequence was inserted (Supplementary Tables S2 and S3). gRNA expression vectors targeting *NEAT1* gRNA, SatIII RNA gRNA and nontargeting (NT) controls for dCas13b were constructed by linearizing the pC0043-PspCas13b crRNA backbone with Bbs I (NEB). The gRNA spacer sequences were then inserted using Ligation high Ver.2 (TOYOBO). Detailed target sequences and primers used in this study are provided in Supplementary data (Supplementary Tables S2 and S3).

### Cell culture

The HeLa, HEK293, and HEK293T cell lines were purchased from ATCC and cultured in Dulbecco’s modified Eagle medium (DMEM; Gibco) supplemented with 10% fetal bovine serum (Gibco), 1% MEM Non-Essential Amino Acids Solution (Gibco), and 1% penicillin–streptomycin (Gibco). All cells were cultured at 37°C in a humidified atmosphere with 5% CO_2_.

### Azide-containing unnatural amino acids

AeF was synthesized as described previously, using 4 M hydrogen chloride (Sigma–Aldrich), *N*-(*tert*-butoxycarbonyl)-L-tyrosine (TCI), 1,2-dibromoethane (TCI), and N,N-dimethylformamide (Fujifilm) [28]. AzF and AmF were purchased from Acrotein and MedChemExpress, respectively.

### Purification of 151UAA-GFP

To produce 151AeF-GFP protein, the pET28a-151TAG-GFP and pULTRA-CNF plasmids were co-transformed into *Escherichia coli* NiCo21 (DE3) (NEB), with transformants selected on Lysogeny Broth (LB) agar plates containing 50 µg/ml kanamycin (Nacalai) and 50 µg/ml Spectinomycin (Nacalai). A single colony was inoculated into 6 ml LB and cultured overnight at 37°C. The following day, the pre-culture was transferred into 600 ml LB supplemented with 1 mM AeF. When the culture reached at OD_600_ of 0.6, protein expression was induced with 1 mM isopropyl-β-d-thiogalactopyranoside (IPTG, Biosynth), and the cells were incubated at 30°C for 24 h. Harvested cells were centrifuged at 5000 × *g* for 7 min at 4°C and stored at −80°C. The frozen cells were thawed, resuspended in 30 ml Lysis buffer [50 mM HEPES (DOJINDO), 0.5 M NaCl (Wako), 0.5 mM dithiothreitol (DTT) (Wako), 20 mM imidazole (Sigma–Aldrich), pH 7.6], and sonicated on ice using a VC-505 sonicator (Sonics and Materials). The lysate was centrifuged at 15 000 × *g* for 10 min at 4°C, and the supernatant was filtered through a 0.45 µm filter (Thermo Fisher Scientific) before loading onto a 5 ml HisTrap FF column (Cytiva) using an AKTA Purifier 10 system (GE Healthcare). Proteins were eluted with elute buffer (50 mM HEPES, 0.5 M NaCl, 0.5 mM DTT, and 300 mM imidazole, pH 7.6). Eluted fractions were analyzed by 15% sodium dodecyl sulphate–polyacrylamide gel electrophoresis (SDS–PAGE), and those containing the target protein were pooled. The pooled fractions underwent overnight dialysis at 4°C in dialysis buffer (50 mM HEPES, 0.15 M NaCl, 0.5 mM DTT) using Spectra/Por 3 dialysis tubing (Repligen). After dialysis, 151AeF-GFP was concentrated using Amicon Ultra-4 10kDa centrifugal filters (Millipore) at 7500 × *g* at 4°C. Protein concentration was determined with a Nanodrop One spectrophotometer (Thermo Fisher Scientific), and purity was assessed via 15% SDS–PAGE. The purified protein was stored at −80°C.

The same procedure was followed to produce 151AzF-GFP and 151AmF-GFP, with slight modifications. For 151AzF-GFP, 1 mM AzF was added to the culture medium in place of AeF. Similarly, for 151AmF-GFP, 1 mM AmF was used. Induction of protein expression was initiated by adding IPTG at OD_600_ = 0 for 151AzF-GFP and at OD_600_ = 0.6 for 151AmF-GFP. All subsequent steps remained unchanged from the standard procedure.

### Formation of RNA-guided green fluorescent protein and DNA-guided green fluorescent protein

RNA-guided green fluorescent protein (RGG) and DNA-guided green fluorescent protein (DGG) complexes were formed using SPAAC. Briefly, custom-synthesized guided nucleic acids, including DBCO-modified, NH_2_-modified, and other chemically modified variants, were obtained from Fasmac or Ajinomoto (Supplementary Table S4). For the *in vitro* assay, 59 pmol of 151UAA-GFP and 59 pmol of nucleic acid (Tel26-DBCO-RNA or Tel26-NH_2_-RNA) were mixed in a 1.5 ml tube and incubated at 4°C for 1.5 h to complete the SPAAC reaction. When optimizing the DBCO-RNA molar ratio for RGG formation, the amount of DBCO-RNA was adjusted relative to the fixed 59 pmol of 151 UAA-GFP and the reaction was carried out under the same conditions. For the cellular assay, 200 pmol of 151AmF-GFP, 200 pmol of DBCO-modified, NH_2_-modified, or other chemically modified guided nucleic acid, and 1 U of RNase Inhibitor (Murine, NEB) were combined and incubated at 4°C for 1.5 h.

### *In vitro* target nucleic acids binding assay for RGG and DGG

The binding capacity of RGG and DGG complexes to target nucleic acids was evaluated using an *in vitro* assay. For the target single-strand RNA (ssRNA) binding assay, 151AmF-GFP was mixed with guided nucleic acids (Tel26-DBCO-RNA, Tel26-NH_2_-RNA, Tel26-DBCO-DNA, or Tel26-NH_2_-DNA). An equal volume of target or nontarget nucleic acids (Target RNA [Tel26] or Non-Target RNA[Tel26]) was added to the pre-formed RGG or DGG complexes. The mixture was incubated at room temperature for 20 min, followed by analysis via gel-shift assay on a 15% SDS–PAGE gel to assess RNA binding capacity. For the target DNA binding assay, a similar procedure was performed. 151AmF-GFP was mixed with guided nucleic acids (Tel26-DBCO-RNA, Tel26-NH_2_-RNA, Tel26-DBCO-DNA, or Tel26-NH_2_-DNA), followed by the addition of an equal volume of target or nontarget single-stranded nucleic acids (Target DNA [Tel26] or Non-Target DNA [Tel26]). After a 20-min incubation at room temperature, single-stranded DNA (ssDNA) binding capacity was evaluated using a gel-shift assay on a 15% SDS–PAGE gel. To prepare double-stranded target DNA, 5 μl of 100 μM single-stranded target DNA (Target DNA [Tel26]) and its complementary strand (Target DNA [compDNA, Tel26]) were mixed with 1 μl of 5 M NaCl. The mixture was heated at 95°C for 2 min and then cooled gradually from 72°C to 4°C over 2 h to allow annealing. The annealed product was purified using EconoSpin spin columns (Epoch Life Science) and resuspend in Tris–EDTA (TE) buffer. The mixtures of pre-formed RGG or DGG complexes with double-stranded target DNA were incubated for 20 min at room temperature, followed by analysis via gel-shift assays on a 15% SDS–PAGE gel to evaluate dsDNA binding capacity. All target nucleic acids were custom-synthesized by Fasmac (Supplementary Table S4).

### Delivery of RGG and DGG in mammalian cells for live-cell imaging

To deliver RGG and DGG via electroporation, cells treated with TrypLE Express (Gibco) were washed three times with cold Dulbecco’s phosphate buffered saline (DPBS, Nacalai) by centrifuging at 300 × *g* for 5 min. The cell concentration was then adjusted to 1 × 10^6^ cells/ml in cold DPBS and incubated on ice for 5 min. Next, 200 pmol of RGG or DGG mixtures was combined with 100 μl of cell suspension (1 × 10^6^ cells/ml) and transferred immediately into a cold 0.2 cm cuvette (Bio-Rad). Electroporation was performed using the Gene Pulser Xcell Electroporation System (Bio-Rad) with the following settings: 110 V, 25 ms pulse duration. Following electroporation, 400 μl of pre-warmed culture medium was added, and the mixture was seeded into one well of an eight-well chamber on cover glass (SCC-008, MATSUNAMI) pre-coated with collagen (IPC-50, Koken) for live-cell imaging. For Lipofectamine CRISPRMAX (Invitrogen) and *Trans*IT (Takara) delivery, RGG and DGG were transfected according to the manufacturer’s instructions with slight modifications. Briefly, 200 pmol of RGG was mixed with the respective transfection reagents as per the manufacturer’s instructions, and the entire mixture was added to the cells.

### Generation of NONO-mScarlet-I3 knock-in cell lines

To generate the NONO-mScarlet-I3 knock-in cell lines, we knocked-in mScarlet-I3 at *NONO* locus of HEK293 cells. To this end, HEK293 cells were seeded in a 10 cm dish. Each 4 μg of pCAG-1BPNLS-Cas9-1BPNLS-2AGFP, NONO gRNA and NONO-mScarlet-I3 donor plasmids were co-transfected into the cells using Lipofectamine 3000, according to the manufacturer’s instructions. Two days after transfection, green fluorescent protein (GFP)-positive cells were sorted using a cell sorter (SH800ZFP, SONY). Approximately 500 cells were seeded in a 10 cm dish, and single colony isolation was performed 12 days after sorting. To ensure that the recovered cells were knocked-in, genome was extracted using the DNeasy Blood and Tissue Kit (Qiagen) following the manufacturer’s protocol. Genomic polymerase chain reaction (PCR) was performed using PrimeSTAR GXL DNA polymerase to identify cells with successful knock-in events using validation of knock-in primers (Supplementary Table S3) to confirm successful integration. Of note, NONO has been well-established as a nuclear marker for *NEAT1* RNA localization based on prior RNA-FISH studies [29]. The use of NONO as a fluorescently tagged reporter in this study is supported by its strong co-localization with *NEAT1*, as demonstrated in the published work [6].

### Generation of HSF1-mScarlet-I3 reporter cell lines using lentivirus

To generate the HSF1-mScarlet-I3 reporter cell lines, a reporter lentivirus was produced. HEK293T cells were seeded in an eight-well plate, and 0.25 μg of pMD2.G, 0.5 μg of pMDLg/pRRE, and 1 μg of pLenti-mScarlet-I3-HSF1 were co-transfected into the cells using Lipofectamine 3000, according to the manufacturer’s manual. Two days post-transfection, the culture supernatant was collected, filtered through a 0.45 μm polyethersulfone filter (Thermo Fisher Scientific), and used for infection. HeLa or HEK293 cells were plated onto one well of a eight-well plate the day before infection, and 600 µl of lentivirus-containing medium was added with 4 µg/ml Polybrene (Tocris Bioscience). Four days post-infection, red-positive cells were sorted using a BD FACSAria Fusion cell sorter (BD Biosciences). Approximately 500 cells were seeded in a 10 cm dish, and single colony isolation was performed 13 days after sorting. The resulting cell lines were selected based on stable nucleus shape and red fluorescence. Of note, HSF1 has previously been used as a nuclear marker for SatIII RNA, based on its strong co-localization observed in RNA-FISH and immunostaining study [30].

### Live-cell imaging of *NEAT1* long noncoding RNA in HEK293 cells

To visualize *NEAT1* using RGG and DGG, HEK293-NONO-mScarlet-I3 reporter cells were electroporated with RGG or DGG and seeded into an eight-well chamber slide with a collagen-coated cover glass. Before imaging, the cells were stained with NucBlue Live ReadyProbes Reagent (Invitrogen), following the manufacture’s instructions. Approximately 36 h post-electroporation, cells were observed using a confocal microscope (Zeiss LSM800 with Airyscan). For visualization of *NEAT1* with dCas13b, we followed the previously reported protocols [6, 31]. In brief, HEK293-NONO-mScarlet-I3 cells were seeded in a 12-well plate and transfected with 0.7 μg of gRNA and 0.3 μg of dCas13b plasmid using Lipofectamine 3000, according to the manufacturer’s instructions. The following day, cells were selected with 1 μg/ml of puromycin (InvivoGen) for 48 h. Puromycin-resistant cells were then reseeded onto an eight-well chambered cover glass coated with collagen. The next day, cells were stained with NucBlue ReadyProbes Reagent and imaged using a confocal microscope. To visualize *NEAT1* with FITC-RNA, 200 pmol of FITC-labelled RNA (Fasmac, Supplementary Table S4) was mixed with 100 μl of 1 × 10^6^ cells/ml HEK293-NONO-mScarlet-I3 reporter cells and electroporated following the same protocol as for RGG. Sixteen hours post-electroporation, cells were observed using a confocal microscope.

### RNA FISH for *NEAT1* long noncoding RNA imaging

Wild-type HeLa cells were electroporated with RGG and plated into a collagen-coated eight-well chamber slide (IWAKI, 5732-008). Prior to fixation, the cells were incubated at room temperature for 30 min. Twenty-four hours post-transfection, the cells were washed three times with ice-cold DPBS and fixed with 4% paraformaldehyde in DPBS (Nacalai) at room temperature for 10 min. The bottom glass slide was detached from the chamber, washed twice with ice-cold DPBS, and permeabilized with 0.1% Triton X-100 (Nacalai) for 12 min. After permeabilization, the samples were washed in 10% formamide (Sigma–Aldrich) in 2 × saline–sodium citrate (SSC: 300 mM NaCl, 30 mM trisodium citrate dihydrate, Wako) and hybridized at 37°C for 16 h with 20 nM of Quasar 570-labelled NEAT1 probes (Stellaris, SMF-2036-1) in 10% dextran (Nacalai). Following hybridization, the samples were incubated in 10% formamide in 2 × SSC at 37°C for 30 min, followed by a 5-min wash in 2 × SSC. After blocking in 3% Normal Goat Serum (Abcam, ab7481) in Tris-buffered saline with Tween-20 (TBST) [137 mM NaCl, 2.7 mM KCl (Wako), 25 mM Tris (Wako), 0.05% Tween-20 (Anatrace), pH 7.4] for 30 min, the cover glass was incubated overnight at 4°C with GFP antibody (Aves, GFP-1020, 1:500) diluted in 3% Normal Goat Serum in TBST. The following day, the samples were washed three times with TBST and incubated with a secondary antibody (goat antichicken 488, Invitrogen, A11039, 1:1000) at room temperature in the dark for 30 min. After washing with TBST, the cells were mounted using ProLong Diamond Antifade Mountant with 4′,6-diamidino-2-phenylindole (DAPI) (Invitrogen) and imaged using a confocal microscope.

### Cell cycle assay

Two days after electroporation, cells were washed twice with DPBS. Subsequently, a propidium iodide (PI) staining solution—comprising 50  µg/ml PI (Sigma, P-4170), 0.25 mg/ml RNase A (Sigma, P-4875), 0.1% trisodium citrate dihydrate, and 0.2 % NP-40 (Nacalai, 25223-04)—was added to the cell suspension. The samples were then incubated at 4°C for 30 min, followed by incubation at 37°C for an additional 30 min. Cell clumps were removed using a 35 µm nylon mesh cell strainer snap cap (Falcon), and the cell cycle profile was analyzed using CytoFLEX S (Beckman Coulter) [32].

### Live-cell imaging of SatIII RNA with RGG

HSF1-mScarlet-I3 reporter HEK293 or HeLa cells were electroporated with RGG. To induce stress, sodium arsenate (SA; Supelco) was added to the electroporated cells at a concentration of 100 μM for 1 h for HEK293 or 300 μM for 1 h for HeLa cells. After treatment, the cells were washed three times with DPBS and incubated in fresh medium at 37°C for 1 h. Subsequently, the cells were stained with NucBlue Live ReadyProbes Reagent, following the manufacture’s instructions. Approximately 36 h post-electroporation, the cells were observed using a confocal microscope.

### RNA FISH for SatIII RNA imaging

Wild-type HeLa cells were electroporated with RGG and plated into one well of an eight-well chamber slide pre-coated with collagen. To induce stress, cells were treated with SA at a concentration of 150 μM for 1 h. Following the treatment, the cells were washed three times with DPBS and incubated in fresh medium at 37°C for 1 h. Twelve hours post-transfection, the cells were washed three times with ice-cold DPBS and fixed in 4% paraformaldehyde in DPBS at room temperature for 10 min. The bottom slide glass was detached from the eight-well chamber, washed twice with ice-cold DPBS, and permeabilized in chilled 70% ethanol (Wako) at 4°C for 1 h. After permeabilization, the samples were washed in 10% formamide in 2 × SSC (300 mM NaCl, 30 mM trisodium citrate dihydrate) and hybridized at 37°C for 16 h with a mixture of each 5 µg/ml of two Cy3-labelled SatIII RNA antisense oligonucleotide probes (Fasmac; Supplementary Table S4) in 10% dextran. Following hybridization, the samples were incubated in 10% formamide in 2 × SSC at 37°C for 30 min, followed by a 5-min wash in 2 × SSC. After blocking in 3% bovine serum albumin (BSA; Wako) in TBST for 30 min, the cover glass was incubated overnight at 4°C with GFP antibody (Aves, GFP-1020, 1:500) in 3% BSA in TBST. The next day, the samples were washed three times with TBST, and incubated with a secondary antibody (goat antichicken 488, Invitrogen, A11039, 1:1000) in the dark at room temperature for 30 min. After washing with TBST, the cells were mounted with ProLong Diamond Antifade Mountant with DAPI. The samples were then imaged using a confocal microscope.

### RNA immunoprecipitation quantitative PCR

Wild-type HeLa cells (6 × 10^5^ cells) were electroporated with 600 pmol of RGG. After 36 h, the cells were subjected to stress treatment with SA at a concentration of 150 µM for 1 h. Following treatment, the cells were washed three times with DPBS and incubated at 37°C for 1 h in fresh medium. The cells were then rinsed twice with ice-cold DPBS, harvested using TrypLE Express, and pelleted by centrifuging at 300 × *g* for 5 min. To isolate nuclei, the cell pellet was resuspended in 1 ml DPBS and combined with 8 ml of Nuclear isolation Buffer [320 mM sucrose (Wako), 10 mM Tris, 5 mM MgCl_2_ (Wako), 1% Triton X-100]. The mixture was incubated on ice for 20 min with intermittent mixing. following by centrifugation at 2 500 × *g* for 15 min. The nuclear pellet was collected and resuspended in 1 ml of RNA immunoprecipitation (RIP) Buffer [150 mM KCl, 25 mM Tris, 5 mM ethylenediaminetetraacetic acid (DOJINDO), 0.5 mM DTT, 0.5% NP40 (Nacalai), 100 U/ml SUPERase-in RNase inhibitor (Invitrogen), Protease Inhibitor Cocktail (Abcam), pH 7.4]. The nuclear lysate was homogenized using a Dounce homogenizer and clarified by centrifuged at 13 000 × *g* for 10 min at 4°C. Half of the supernatant was incubated overnight at 4°C with 6 μg of GFP antibody (Abcam, ab290). The samples were then incubated with 25 μl of Dynabeads Protein G (Invitrogen) for 2 h at 4°C. The beads were washed three times with 500 μl of RIP Buffer and once with 500 μl of phosphate buffered saline, followed by centrifugation at 2 500 × *g* for 1 min at 4°C. To extract RNA, the beads pellets were processed using TRIzol RNA extraction reagent, following the manufacture’s protocol. The extracted RNA was treated with DNase I (NEB) to remove genomic DNA and reverse-transcribed into cDNA using the SuperScript IV First-Strand Synthesis System, according to the manufacture’s instructions. Quantitative PCR (qPCR) was performed using SYBR Green Realtime PCR Master Mix (TOYOBO) and a CFX384 Touch Real-Time PCR Detection System (Bio-Rad). The qPCR signal for each target transcript was normalized to GAPDH mRNA as an internal reference control and expressed as percentage of input (% input). This represents the proportion of target RNA recovered by immunoprecipitation relative to the total RNA in the corresponding lysate prior to immunoprecipitation, reflecting relative enrichment rather than absolute RNA abundance. Primers used for qPCR are listed in Supplementary Table S3.

### Image acquisition and analysis

Electrophoresis images were acquired using the Gel Doc XR + Gel Documentation System (Bio-Rad) and analyzed with Image Lab 6.0 (Bio-Rad). Confocal microscopic images were captured using an LSM800 with Airyscan and processed with Zen 2.6 (blue edition) software (Zeiss). For spot count analysis, individual fluorescent foci corresponding to RGG, reporter proteins, or RNA FISH probes were manually identified and quantified within nuclear regions using ZEN software. For fluorescence intensity measurements, the integrated green fluorescence intensity within red-labelled foci was quantified using ZEN software, without background subtraction.

### Statistical analysis

Data were analyzed using Graphpad Prism 8 (Graphpad Software). For comparisons involving two or more groups, one-way analysis of variance (ANOVA) followed by Dunnett’s multiple comparisons test or the Kruskal–Wallis test followed by Dunn’s multiple comparisons test was used. For comparisons between two groups, either Student’s *t*-test or the Mann–Whitney test was applied, depending on data-distribution. Statistical significance was defined at *P *<.05.

## Results

### Development of chemically conjugated DNA/RNA–protein complexes

**Figure 1.**
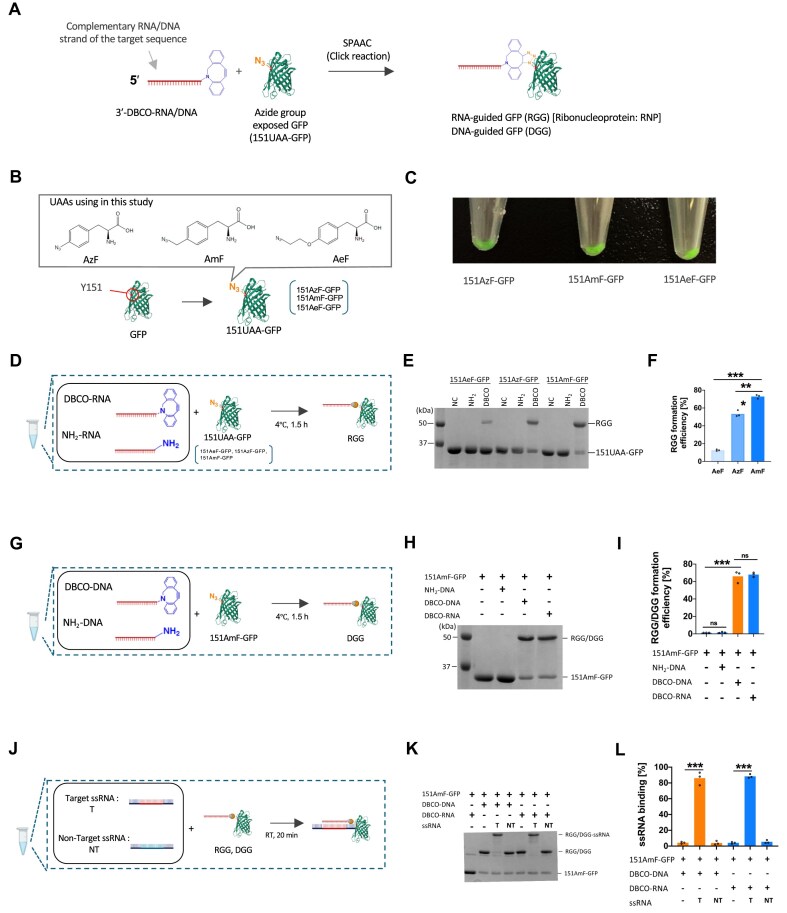
Design, preparation, and evaluation of RGG and DGG for target ssRNA binding. (**A**) Schematic illustration of RGG and DGG. (**B**) Chemical structure of the UAA used and illustration of azide-exposed GFP used for RGG and DGG preparation. AzF, *p*-azido-L-phenylalanine. AmF, *p*-azidomethyl-L-phenylalanine. AeF, *p*-azidoethoxy-L-phenylalanine. 151AzF-GFP, 151AmF-GFP, and 151AeF-GFP denote GFP variants with these UAAs incorporated at tyrosine 151. (**C**) Fluorescence images of purified 151UAA-GFPs variants. (**D**) Workflow illustrating RGG preparation. (**E**) Gel shift analysis demonstrating the conjugation of 151UAA-GFPs with DBCO-RNA. (**F**) Quantitative analysis of conjugation efficiency shown in panel (E). Statistical significance was determined using one-way ANOVA followed by Dunnett’s multiple comparisons test (*n *= 3, ***P <.001). (**G**) Workflow illustrating DGG preparation. (**H**) SDS–PAGE analysis demonstrating the conjugate of 151AmF-GFP and DBCO-DNA. (**I**) Quantitative analysis of conjugation efficiency shown in panel (**H**). Statistical significance was determined using one-way ANOVA followed by Dunnett’s multiple comparisons test (*n *= 3, ***P <.001, ns: not significant). (**J**) Workflow illustrating ssRNA binding evaluation of RGG and DGG. (**K**) Gel shift analysis demonstrating the specific ssRNA binding capacity of RGG and DGG. T: target ssRNA. NT: nontarget ssRNA. (**L**) Quantitative analysis of ssRNA binding shown in panel (**K**). Statistical significance was determined using one-way ANOVA followed by Dunnett’s multiple comparisons test (*n *= 3, ***P <.001).

To construct synthetic DNA/RNA–protein complexes, we employed SPAAC to conjugate DBCO and azide groups. DBCO-functionalized DNA (DBCO-DNA) and RNA (DBCO-RNA) were utilized as guide nucleic acids for specific sequences targeting, while GFP was selected as the functional protein to facilitate intracellular localization. To ensure nuclear localization, GFP was engineered with two bipartite nuclear localization signals at both its N- and C-termini (Supplementary Table S1) [33]. For SPAAC-mediated conjugation, azide groups were introduced onto the GFP surface via a site-specific incorporation of unnatural amino acid (UAA) [34–36]. This technique uses engineered orthogonal transfer RNA (tRNA) and tRNA synthetase pairs to substitute codons with azide-containing UAAs, enabling precise chemical modifications (Supplementary Fig. S1A) [37, 38]. By combining DBCO-functionalized nucleic acids with azide-modified GFP, we attempted to form specific and stable RGG and DNA-guided GFP (DGG) complexes (Fig. 1A). These conjugates represent a simple approach to creating programmable, sequence-specific protein tools for applications such as nucleic acid targeting.

Using established UAA incorporation methods, we designed a GFP variant, 151UAA-GFP, where an azide-containing UAA replaced the tyrosine residue at position 151 (Supplementary Table S1). This position was previously identified as suitable for azide modification without compromising GFP fluorescence [34–36, 39]. To enhance azide group accessibility and optimize conjugation efficiency, we tested three UAAs, AzF, AmF, and AeF, which differ in the distance of the azide group from the benzene ring (Fig. 1B). All 151UAA-GFPs variants, including 151AzF-GFP, 151AmF-GFP, and 151AeF-GFP, were successfully purified as full-length proteins and retained green fluorescence (Fig. 1C and Supplementary Fig. S1B). Conjugation efficiency was assessed under SPAAC conditions by reacting 151UAA-GFP with DBCO-RNA. Among the tested variants, 151AmF-GFP demonstrated the highest conjugation efficiency, achieving a conjugation rate of 73% (Fig. 1D–F). The efficiency plateaued at a 1:1 molar ratio of DBCO-RNA to GFP, establishing this ratio as optimal for subsequent experiments (Supplementary Fig. S1C–E). To confirm versatility of the system, we generated DGG by reacting 151AmF-GFP with DBCO-DNA. The conjugation efficiency for DGG was comparable to RGG (Fig. 1G–I).

Specificity analyses revealed that both RGG and DGG complexes selectively bound their target ssRNA with high efficiencies of 88% and 86%, respectively (Fig. 1J–L). Notably, both complexes exclusively recognized ssDNA without binding to double-stranded DNA (dsDNA), underscoring their specificity for single-strand templates (Supplementary Fig. S1F–H). This preference is likely due to the inability of the guide strand to invade the stable duplex structure of dsDNA under physiological conditions. These findings demonstrate the successful development of chemically conjugated DNA and RNA–protein complexes with sequence and strand specificity for ssRNA and ssDNA, providing a programmable tool for sequence-specific nucleic acid recognition and modification.

### Intracellular *NEAT1* RNA imaging with RGG

Building on the *in vitro* success of RGG and DGG in binding target ssRNA, we evaluated the ability of RGG to bind endogenous ssRNA within cells. *NEAT1*, well-characterized nuclear-localized long noncoding RNA (lncRNA) involved in paraspeckle formation and widely studied in RNA imaging [6, 40], was selected as the target. To visualize *NEAT1*, we established HEK293 knock-in reporter cells in which the *NEAT1*-binding protein NONO was endogenously tagged with mScarlet-I3, taking advantage of its known co-localization with *NEAT1* [41, 42] (Supplementary Fig. S2A–D). The fidelity of this reporter system was validated using the established dCas13b-3xEGFP (dCas13b) RNA-targeting system, which showed clear co-localization with NONO foci, as previously reported [6, 40] (Supplementary Fig. S2E). To target *NEAT1*, DBCO-RNA complementary to the *NEAT1* sequence was conjugated to 151AmF-GFP *in vitro* to form *NEAT1*-specific RGG. For intracellular delivery, we compared electroporation with two commercial ribonucleoprotein (RNP) transfection reagents, Lipofectamine CRISPRMAX, and *Trans*IT. Electroporation emerged as the most efficient method for introducing RGG into wild-type HEK293 cells, outperforming the commercial reagents (Supplementary Fig. S2F), and was therefore chosen for all subsequent experiments.

**Figure 2.**
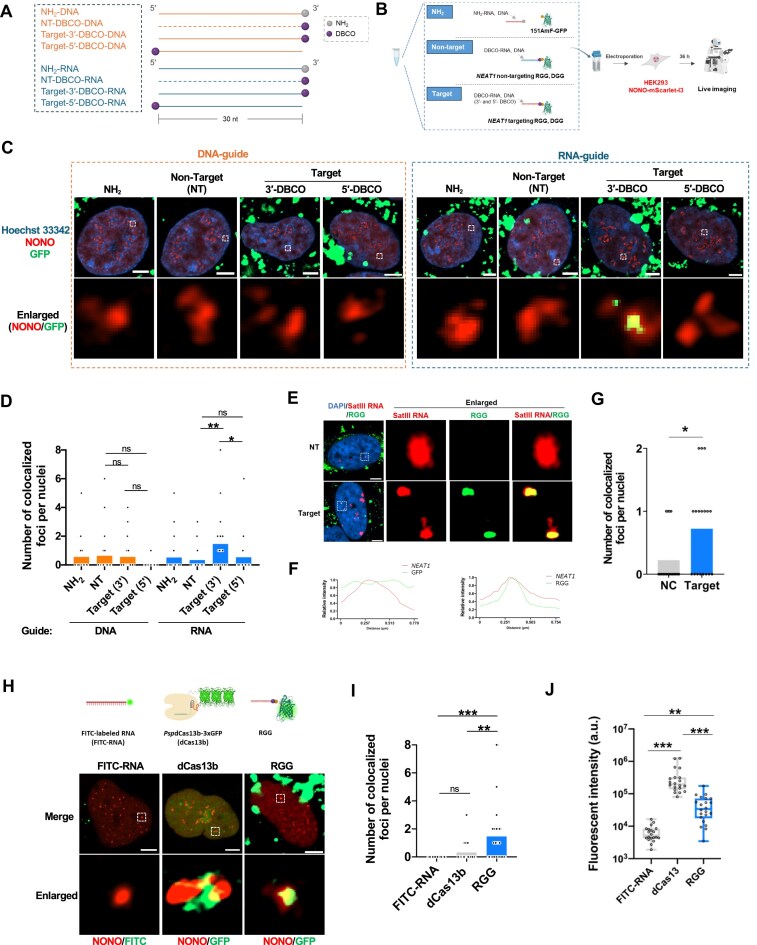
Live-cell imaging of *NEAT1* using DGG and RGG. (**A**) Schematic representation of guide nucleic acids for DGG and RGG. The *NEAT1* target sequence is shown as solid lines, while the nontarget sequence is represented by dotted lines. (**B**) Workflow depicting the process for live-cell imaging of *NEAT1* using DGG and RGG. (**C**) Representative fluorescent images of HEK293-NONO-mScarlet-I3 cells introducing DGG (left) or RGG (right). *NEAT1* localization is visualized with NONO-mScarlet-I3 (red), GFP signals from DGG/RGG (green), and Hoechst33342 (blue). NT: nontarget. Scale bars: 5 μm. (**D**) Quantitative analysis of NONO co-localization with DGG or RGG signals shown in panel (**C**). Statistical analysis was performed using the Kruskal–Wallis test followed by Dunn’s multiple comparisons test (NH_2_-DNA: *n *= 25, NT-DNA: *n *= 25, Target-3′-DBCO-DNA: *n *= 25, Target-5′-DBCO-DNA: *n *= 20, NH_2_-RNA: *n *= 24, NT-RNA: *n *= 41, Target-3′-DBCO-RNA: *n *= 39, Target-5′-DBCO-RNA: *n *= 39, *P <.05, **P <.01, ***P <.001, ns: not significant). (**E**) Representative RNA-FISH images of HeLa cells following RGG transfection. The top panels show NH_2_–RNA and GFP signals in the negative control (NC), while the bottom panels depict *NEAT1*-targeting RGG. Scale bars: 5 μm. (**F**) Line profile analysis of the RNA-FISH images shown in panel (E), comparing NC (left) and RGG (right). Red (*NEAT1*) and green (RGG/GFP) signal intensity plots demonstrate spatial co–localization. (**G**) Quantitative analysis of *NEAT1* co-localization with RGG in HeLa cells. Statistical analysis was performed using the Mann–Whitney test (NC: *n *= 18, Target: *n *= 18, *P <.05). (**H**) Representative fluorescence images showing NONO co-localization with FITC-labelled RNA probe, dCas13b, and RGG. Scale bars: 5 μm. (**I**) Quantification of NONO co-localization with the RNA imaging tools shown in panel (H). Statistical analysis was performed using the Kruskal–Wallis test followed by Dunn’s multiple comparisons test (FITC-RNA: *n *= 37, dCas13b: *n *= 28, RGG: *n *= 39, **P <.01, ***P <.001, ns: not significant). (**J**) Quantitative analysis of green fluorescence intensity from the RNA imaging tools is shown in panel (H). Statistical analysis was performed using the Kruskal–Wallis test followed by Dunn’s multiple comparison test (FITC-RNA: *n* = 20, dCas13b: *n* = 20, RGG: *n* = 22, **P <.01, ***P <.001).

*NEAT1*-specific RGG and DGG were introduced into HEK293 reporter cells via electroporation, alongside controls that included nontarget (NT) DBCO-RNA and DBCO-DNA conjugated with 151AmF-GFP to form NT-RGG and NT-DGG, as well as NH_2_-modified RNA and DNA, which cannot conjugate with azide groups (Fig. 2A). Live-cell imaging performed 36 h post-introduction revealed that DGGs, regardless of 5′- or 3′-DBCO placement, failed to specifically image *NEAT1* (Fig. 2B–D). In contrast, RGGs with 3′-DBCO-modified RNA clearly visualized *NEAT1* foci, showing specific co-localization with NONO (Fig. 2B–D and Supplementary Fig. S2G). Conversely, RGGs with 5′-DBCO-modified RNA exhibited diffuse and nonspecific fluorescence patterns that were indistinguishable from those observed with NT controls, indicating a loss of target specificity. These findings highlight the critical role of 3′-end modification for efficient and accurate RNA imaging with RGG. To further confirm that the observed signal indeed reflected binding to *NEAT1* lncRNA rather than to the NONO protein, we performed RNA FISH. The RGG signal co-localized with the FISH probe signal for *NEAT1*, confirming that RGG directly targets *NEAT1* lncRNA (Fig. 2E–G).

To assess whether using multiple gRNAs could improve labelling efficiency, we designed an additional 3′-DBCO-modified RNA (gRNA2) targeting a distinct site on *NEAT1*. However, gRNA2 yielded a markedly weaker signal than the original guide (gRNA1), and their combined use did not improve labelling efficiency, instead resulting in reduced performance compared to gRNA1 alone (Supplementary Fig. S2H). These results indicate that guide selection is critical for efficient RNA labelling, and that simply increasing the number of target sites does not necessarily improve performance. The evaluation whether RGG introduction perturbs cellular function, we performed a cell–cycle assay. Compared to control (empty–electroporated) cells, RGG–introduced cells showed no significant changes in cell–cycle distribution (Supplementary Fig. S2I). These results suggest that RGG does not significantly perturb cell-cycle progression, indicating minimal cellular impact under the tested conditions.

To benchmark the performance of RGG, we compared it with other two *NEAT1* imaging tools, including FITC-labelled RNA probe and dCas13b [6, 43]. For consistency, all tools used the same guide sequence (Supplementary Tables S2–S4). Among these, RGG demonstrated the highest co-localization efficiency with NONO (Fig. 2H and I). The FITC-labelled RNA displayed minimal green fluorescence in the nucleus, while dCas13b produced fewer co-localized signals despite having the highest fluorescence intensity at the foci (Fig. 2J). Furthermore, dCas13b was overexpressed via transfection of a genetically encoded plasmid, leading to the formation of aggregates in cells, as reported previously [6] (Supplementary Fig. S2J). These findings establish RGG with 3′-DBCO-modified RNA as a robust and specific tool for live-cell *NEAT1* RNA imaging.

### Optimizing imaging efficiency of RGG

**Figure 3.**
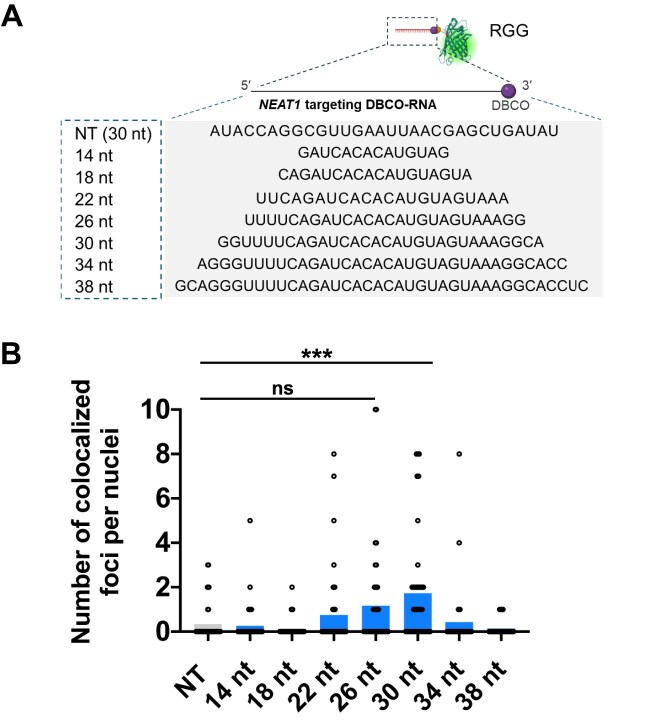
Optimization of DBCO-RNA for *NEAT1* imaging using RGG. (**A**) Schematic representation illustrating the optimization of DBCO-RNA length for *NEAT1* imaging. (**B**) Quantitative analysis of NONO co-localization with RGG conjugated to DBCO-RNA of varying lengths. Statistical analysis was performed using the Kruskal–Wallis test followed by Dunn’s multiple comparisons test (NT: *n *= 30, 14 nt: *n *= 41, 18 nt: *n *= 45, 22 nt: *n *= 44, 26 nt: *n *= 41, 30 nt: *n *= 41, 34 nt: *n *= 35, 38 nt: *n *= 35, ***P <.001, ns: not significant)

To boost the imaging capability of RGG, we systematically optimized the length of the RNA guide. Specially, we designed DBCO-RNAs ranging from 14 to 38 nucleotides targeting *NEAT1* and evaluated their performance (Fig. 3A). Among these, the 30 nt DBCO-RNA demonstrated a significant increase in co-localization efficiency compared to the NT RGG control (Fig. 3B and Supplementary Fig. S3A). Although the 26 nt DBCO-RNA exhibited a similar trend, the improvement was not statistically significant.

To further optimize imaging performance, we explored additional chemical modifications known to enhance nucleic acid stability and nuclease resistance, including phosphorothioate (PS), 2′-O-methoxyethyl (2′-MOE), locked nucleic acids, inverted thymidine (InvT), and evopreQ_1_ (a modified prequeosine_1_-1 riboswitch aptamer forming a stable pseudoknot) (Supplementary Fig. S3B–E) [44–51]. Partially modified 30-nt DBCO-RNAs were categorized based on the applied modification, with fully PS- or 2′-MOE-modified sequences referred to as PS_all_ and 2′-MOE_all_, respectively. The modified DBCO-RNAs, such as PS_all_ and 2′-MOE, demonstrated significantly higher imaging efficiency compared to the NT control (Supplementary Fig. S3D and E). However, quantitative analysis of NONO co-localization with RGG conjugated to chemically modified DBCO-RNAs showed only a trend toward improved performance over unmodified DBCO-RNA, without reaching statistical significance.

Efforts to adapt DNA-guided DGG for *NEAT1* imaging proved unsuccessful, regardless of variations in DBCO-DNA length or additional chemical modifications (Supplementary Fig. S3F–K). Although PS_all_-modified DBCO-DNA showed substantial improvement over the NT control, it did not outperform unmodified DBCO-DNA. These findings identify 30-nt DBCO-RNA as the optimal guide length for *NEAT1* imaging with RGG. Furthermore, they demonstrate that *NEAT1* imaging can be achieved without additional chemical modifications for nuclease resistance, underscoring the robustness of unmodified DBCO-RNA for this application.

### Intracellular RNA targeting with RGG for SatIII RNA visualization in multiple cell lines

Building on the successful intracellular imaging of *NEAT1* lncRNA using RGG, we next evaluated its ability to target additional nuclear-localized ssRNA, focusing on Satellite III RNA (SatIII RNA) and its applicability across different cell types. SatIII RNA is a nuclear lncRNA with repetitive sequences, actively transcribed during stress conditions such as heat shock or UV exposure [30, 52]. It remains localized at transcription sites through interaction with the transcription activation factor HSF1 [30, 52]. To visualize SatIII RNA, we established HEK293 and HeLa reporter lines expressing HSF1 fused with mScarlet-I3. HSF1 is widely used as a proxy marker for SatIII RNA owing to its spatial co-localization during stress responses [6, 53] (Supplementary Fig. S4A and B). The specificity of this reporter was validated using the dCas13b, which robustly labelled SatIII RNA (Supplementary Fig. S4C and D), in agreement with prior report [6].

**Figure 4.**
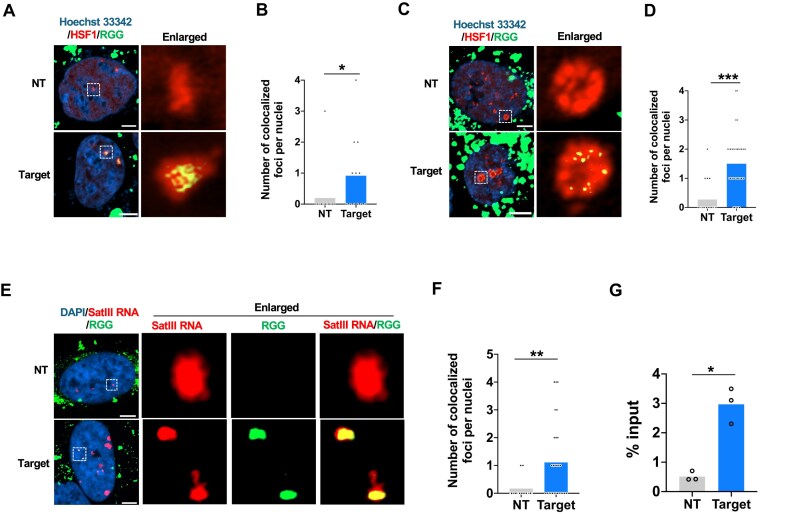
Intracellular SatIII RNA imaging with RGG in live cells. (**A**) Representative fluorescence images showing RGG introduced into live HEK293-HSF1-mScarlet-I3 cells following SA treatment (100 μM for 1 h). The images depict NT RGG (top) and SatIII RNA targeting RGG (bottom). NT: nontarget. Scale bars: 5 μm. (**B**) Quantitative analysis of NSF1 co-localization with RGG in HEK293-HSF1-mScarlet-I3 cells. Statistical analysis was performed using the Mann–Whitney test (NT: *n *= 35, Target: *n *= 22, *P <.05). (**C**) Representative fluorescence images of RGG introduced into live HeLa-HSF1-mScarlet-I3 cells after SA treatment (300 μM for 1 h). The images depict NT RGG (top) and SatIII RNA targeting RGG (bottom). NT: nontarget. Scale bars: 5 μm. (**D**) Quantitative analysis of HSF1 co-localization with RGG in HeLa-HSF1-mScarlet-I3 cells. Statistical analysis was performed using the Mann–Whitney test (NT: *n *= 40, Target: *n *= 38, ***P <.001). (**E**) Representative RNA FISH images of HeLa cells treated with SA (150 μM for 1 h) after RGG transfection. The images depict NT RGG (top) and SatIII RNA targeting RGG (bottom). NT: nontarget. Scale bars: 5 μm. (**F**) Quantitative analysis of SatIII RNA co-localization with RGG in HeLa cells. Statistical analysis was performed using the Mann–Whitney test (NT: *n *= 35, Target: *n *= 27, **P <.01). (**G**) Quantitative analysis of RIP-qPCR data for RGG-transfected HeLa cells treated with SA (150 μM, 1 h). The y-axis indicates the percentage of total RNA recovered from each RGG pull-down. Statistical analysis was performed using Student’s *t*-test (*n *= 3, *P <.05).

For RGG preparation, DBCO-RNA complementary to the SatIII RNA repeat sequence was conjugated to 151AmF-GFP *in vitro*. This SatIII RNA-specific RGG was introduced into HEK293 reporter cells via electroporation, followed by live-cell imaging 36 h post-introduction. Imaging confirmed specific co-localization of RGG to HSF1 (Fig. 4A and B). To evaluate the versatility of RGG across cell lines, the complex was introduced into HeLa reporter cell. Similar results were observed, with clear co-localization of RGG and HSF1 in nuclear foci (Fig. 4C and D). Comparison with the conventional dCas13 imaging system revealed comparable labelling efficiency for SatIII RNA between RGG and dCas13 (Supplementary Fig. S4E). To further validate the specificity of RGG for SatIII RNA, RNA FISH was performed 12 h after RGG electroporation, confirming co-localization of RGG with SatIII RNA, enabling rapid visualization (Fig. 4E and F and Supplementary Fig. S4F). Time-course analysis showed that RGG signals became detectable as early as 6 h after delivery, but progressively declined, becoming indistinguishable from the NC by 48 h (Supplementary Fig. S4G and H). Additionally, RIP followed by qPCR (RIP-qPCR) was used as a nonimaging approach to verify target specificity. RGG introduced into wild-type HeLa cells exhibited strong enrichment for SatIII RNA compared to controls, further corroborating its specificity (Fig. 4G). These results establish RGG as a reliable and versatile tool for targeting nuclear-localized ssRNA across multiple cell lines. Its specificity and adaptability highlight its potential as a robust platform for RNA imaging and molecular biology applications.

## Discussion

The CRISPR-Cas system, originally discovered as a prokaryotic immune defence in bacteria and archaea, elegantly guides proteins to specific nucleic acid sequences using RNA molecules [54]. Inspired by this natural system, we sought to develop a simple yet effective method for RNA–protein and DNA–protein conjugation to target specific sequences within cells. To achieve this, we chemically linked RNA to GFP and DNA to GFP, creating RGG and DGG, respectively. The creation of RGGs and DGGs relies on a click reaction, a class of highly efficient and specific chemical reactions ideal for linking biomolecules. Specifically, we used SPAAC, a type of click reaction, to form a stable and specific bond between RNA/DNA and protein. In this approach, the ends of RNA and DNA molecules complementary to the target sequence were modified with DBCO, while the protein surface was decorated with azide groups. These two functional groups react spontaneously via SPAAC to create a covalent RNA/DNA–protein complex. Among the various click reactions available, SPAAC was chosen for its safety and practicality. While other methods, such as Copper-catalyzed azide-alkyne 1,3-dipolar cycloaddition (CuAAC) [55, 56] and the Inverse electron-demand Diels–Alder reaction (IEDDA) [57], are widely used, they have notable limitations. CuAAC requires a copper catalyst, which can be toxic in biological systems, while IEDDA involves expensive reagents and limited accessibility. SPAAC, in contrast, is bioorthogonal (i.e. it does not interfere with biological processes), catalyst-free, and operational under mild conditions, making it an ideal choice for constructing RGGs and DGGs.

To optimize the generation of RGGs and DGGs, we further investigated factors influencing their conjugation efficiency via SPAAC. Previous research has shown that the efficiency of conjugating azide-exposed GFP using click reactions depending on the spatial arrangement of the azide group relative to its benzene ring [35]. Consistently, in this study, we observed that the choice of azide compound significantly impacted conjugation efficiency, with 151AmF-GFP achieving the highest efficiency (Fig. 1E and F). We propose that AzF, with its shortest distance between the azide group and benzene, displayed lower reactivity with DBCO-RNA or DBCO-DNA due to steric hindrance from GFP. These findings underscore the critical role of azide compound selection in optimizing the conjugation efficiency of RGGs and DGGs. By addressing structural challenges, this study offers valuable insights into enhancing nucleic acid-protein conjugation strategies, advancing the development of high-precision RNA- and DNA-guided molecular tools.

To enable intracellular RNA visualization, we compared DNA and RNA guides conjugated to GFP via either 5′- or 3′-DBCO modifications. Among these, only the 3′-DBCO-modified RNA effectively directed the DBCO-RNA-linked GFP signal to co-localize with the target RNA, thereby demonstrating functional targeting capability (Fig. 2A–D). The reduced performance of DGGs may be due to RNaseH-mediated degradation of RNA:DNA hybrids [58, 59]. Notably, 5′-DBCO RNA guides failed to function, possibly reflecting differential susceptibility to endogenous exonucleases, with 5′→3′ activity potentially more prominent in cells. The 3′-DBCO RNA guide may also be less susceptible to 3′→5′exonucleases. Although the RNA guides form short duplexes with their targets, which could in principle be recognized by RNase III enzymes such as Dicer [60, 61]—human Dicer is generally thought to reside in the cytoplasm, but has also been reported in the nucleus, where it may contribute to regulating endogenous double-stranded RNA (dsRNA) levels. Given the short length and lack of typical termini in our duplexes, as well as the absence of definitive evidence for nuclear Dicer–mediated cleavage in this context, we consider any Dicer contribution to be possible but unlikely, with RNaseH activity remaining the more plausible primary cause of DGG underperformance. Additionally, thermodynamic studies indicate that RNA:RNA duplexes are more stable than RNA:DNA hybrids [62], which may not be the primary factor but could partially explain why only RGG successfully bound to target RNA in cells. These results suggest that while RGG is well-suited for intracellular RNA imaging, DGG might be appropriate for applications targeting ssDNA, where RNaseH susceptibility is not a concern.

Through a systematic evaluation of gRNA lengths for generating RGG, we identified 30 nt as the optimal length for *NEAT1* imaging in cells (Fig. 3). This finding is consistent with previous studies demonstrating that the gRNAs ranging from 22 to 30 nt provide a significant signal-to-noise ratio in *NEAT1* imaging using dCas13b [6]. These results suggest that around 30 nt is likely an ideal length for specifically binding to target sequences, although the optimal length may vary depending on the target RNA sequence. Previous research has also shown that proteins with an excessively negative charge can be efficiently delivered to human cultured cells [63]. In the case of RGG, the charge varies depending on the length and type of nucleic acid used, which could influence delivery efficiency based on the target sequence. Regarding further modification to enhance nuclease resistance, incorporating phosphorothioate (PS) modifications to all bases of DBCO-RNA/DNA in RGG/DGG showed a trend towards increased RNA imaging efficiency, although the difference was not statistically significant. PS modification alters the phosphate backbone and is known to provide a nuclease resistance [44], which may contribute to the observed trend. To facilitate broader application of the RGG system, we also provide practical guidance for selecting effective guide sequences. While our screen did not identify a universal motif predictive of labeling efficiency, our data suggest that guide sequence selection significantly impacts RGG performance (Supplementary Fig. S2H). Therefore, we recommend using *in silico* design tools such as CHOPCHOP (https://chopchop.cbu.uib.no/) to identify candidate regions with high target accessibility and minimal off-target potential. Notably, our best-performing guide (gRNA1) was computationally designed and overlapped with a previously validated region used for dCas13b-based RNA imaging [6], underscoring the value of informed *in silico* design. These practical insights will help guide users in implementing RGG for diverse RNA targets.

When compared to other imaging tools, the *NEAT1*-targeting RGG demonstrated superior efficiency in RNA imaging for *NEAT1* in our experimental conditions (Fig. 2H and I). This advantage is likely due to its nuclear localization capability and small molecular size. Effective live imaging of nuclear-localized lncRNA, such as *NEAT1*, relies on the addition of an NLS to facilitate proper targeting. Although the FITC-RNA probe has a small molecular weight, it lacks an NLS, which likely hindered its ability to bind and visualize *NEAT1* effectively. On the other hand, while the *NEAT1*-targeting dCas13b-gRNA complex includes an NLS, its large size, at 232 kDa, making it challenging to access *NEAT1. NEAT1* is embedded in a complex nuclear structure involving >40 different proteins [41, 42], which further complicates imaging. In contrast, the *NEAT1*-targeting RGG with a 30 nt DBCO-RNA guide has a molecular weight of only 43 kDa. Its binding mechanism, driven by azide-DBCO chemistry, positions it among the smallest RNA imaging tools using fluorescent proteins. Additionally, RGG avoids nonspecific nucleolar accumulation often observed with CRISPR-Cas-based imaging systems [6, 10], and unlike genetically encoded fluorescent proteins, it does not aggregate in the nucleolus. Its small size and direct labelling chemistry allows rapid imaging—e.g. clear visualization of SatIII RNA was achieved within 6 h of introduction (Supplementary Fig. S4G and H). These features make RGG an promising tool for RNAs imaging that are otherwise challenging to access. While these advantages are clear, it is important to note that high but not absolute co-localization between RNA labelling approaches and RNA-FISH has been consistently reported for *NEAT1* and SatIII RNA. Consistent with our findings (including Figs 2E and 4E), previous studies have also observed incomplete overlap, likely reflecting differences in detection principles, target accessibility, competition, and higher-order RNA structures [6, 53, 64]. Consequently, the observed performance of RGG should be interpreted in the context of the specific experimental conditions tested. We cannot exclude the possibility that other imaging systems may perform better under different contexts. Further systematic comparisons will be needed to fully evaluate the generalizability of RGG’s advantages. Despite these limitations, as a synthetic RNP, RGG is likely to degrade quickly within cells (Supplementary Fig. S4G and H), thereby minimizing off-target effects from the effector protein. This quality, combined with its synthetic design, positions RGG as an ideal platform for RNA localization and functional analysis through live imaging. Furthermore, the chemically synthesizable DBCO-RNA guide offers opportunities for further functionalization. For example, modification with caged compounds could enable light-inducible binding to target RNA sequences, expanding the versatility of RGG for dynamic and spatiotemporal RNA studies [65].

In our current system, we observed cytoplasmic background signals, likely stemming from nonspecific aggregation of unbound GFP-RGG probes. Similar cytoplasmic accumulation has been reported in previous studies using electroporation-based delivery of protein–RNA complexes, such as Cas9 RNPs [66], and is thought to result from local supersaturation of introduced proteins and stress on the cellular protein folding machinery [67]. Although this background did not interfere with detection of nuclear RNA foci in our assays, reducing nonspecific cytoplasmic signals will be important for expanding the system’s utility—particularly for imaging cytoplasmic RNAs. To mitigate background signal, future improvements in probe design will be essential. For example, a split-GFP RGG architecture that reconstitutes fluorescence only upon specific RNA binding, or a thioredoxin-fused GFP-RGG variant with enhanced solubility, may reduce aggregation. We also tested alternative delivery approaches; however, commercially available RNP transfection reagents showed limited efficiency in our hands (Supplementary Fig. S2F). Moving forward, incorporating more advanced delivery platforms—such as lipid nanoparticles—may offer improved control over cytoplasmic delivery and help minimize background signal.

In addition to cytoplasmic aggregation, another potential limitation of the RGG system is the occasional signal observed in NT controls. While these signals were generally diffuse and substantially weaker than those from targeting guides, their presence suggests low-level nonspecific interactions or passive accumulation. This phenomenon is also observed in other RNA imaging modalities, such as CRISPR–dCas13-based platforms [6], and emphasizes the importance of including appropriate controls and optimizing guide design to minimize off-target binding. Careful interpretation is especially warranted when visualizing low-abundance transcripts or subtle localization patterns. Further refinements in probe architecture, delivery methods, and validation using orthogonal techniques such as RNA-FISH may help address this limitation and improve overall specificity.

In conclusion, we introduced a novel synthetic nucleic acid–protein complex by chemically conjugating DNA/RNA to a protein, creating a precise and versatile tool for intranuclear RNA imaging. For researchers seeking to apply this system, we recommend beginning with 30 nt RNA guides bearing a 3′-DBCO group modification, as this configuration showed optimal imaging performance in our experimental evaluations. This innovative approach enabled accurate and efficient visualization of nuclear-localized RNAs, such as *NEAT1* and SatIII RNA. The modular design of RGG further allows for conjugation of various effector proteins in place of GFP, transforming it into a versatile and customizable tool for RNA localization and functional studies. These unique features position our direct conjugation of DNA/RNA and protein as a groundbreaking platform at the forefront of RNA biology, chemical biology, and molecular engineering, unlocking new possibilities for precise nucleic acid recognition and dynamic molecular analysis.

## Supplementary Material

gkaf1147_Supplemental_File

## Data Availability

The data underlying this article is incorporated in the paper and online supplementary material.
