# Supplementary material for: Development of programmable RNA imaging with RNA-guided GFP via click chemistry
Source: Nucleic Acids Res. 2025 Nov 8;53(20):gkaf1147. doi: 10.1093/nar/gkaf1147 (PMC12597101; doi:10.1093/nar/gkaf1147)
Supplement: gkaf1147_Supplemental_File [file gkaf1147_supplemental_file.pdf]

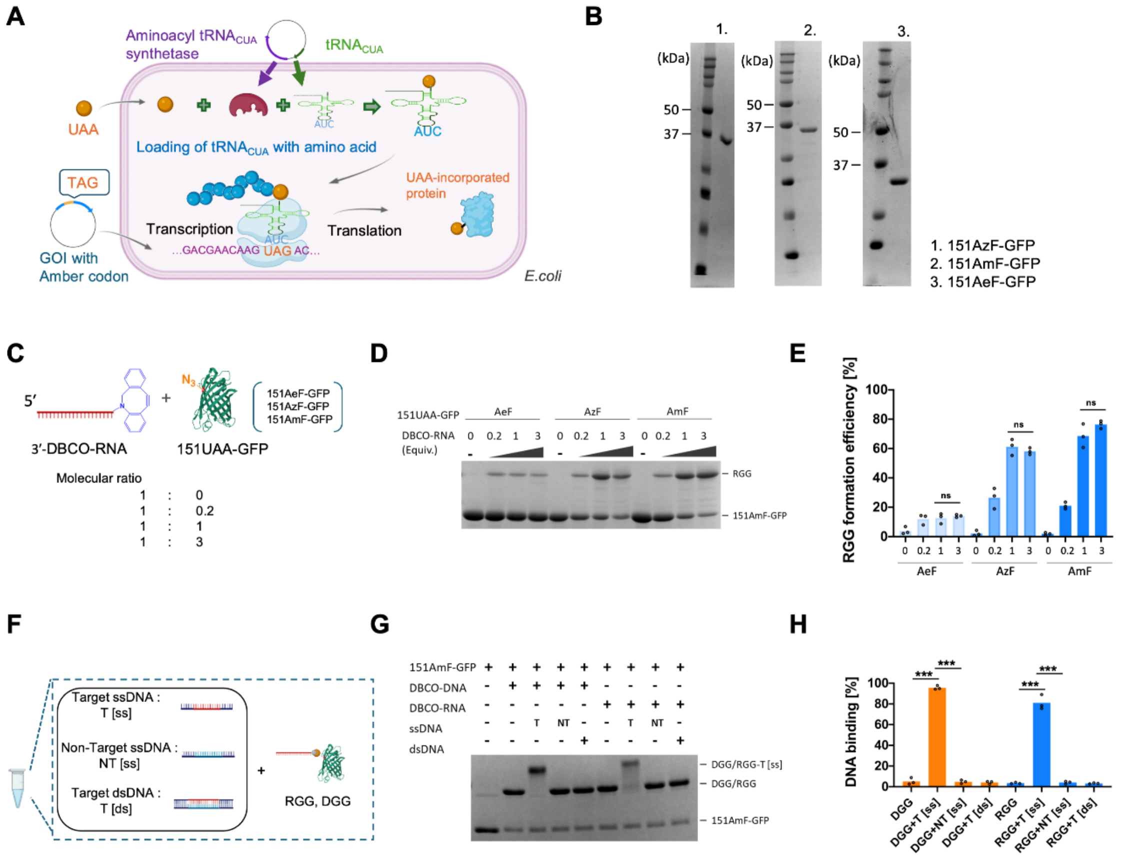

Figure S1: Preparation and evaluation of RGG and DGG. (**A**) Schematic representation of site-specific incorporation of unnatural amino acids (UAAs) in *E. coli*. A UAA, an archaeal orthogonal tRNA/tRNA synthetase pair and a gene of interest (GOI) containing an amber stop codon (TAG) at the desired incorporation site are introduced into *E. coli*. The presence of the UAA enables the production of full-length GOI-derived protein. (**B**) SDS-PAGE analysis showing purified 151UAA-GFP variants. (**C**) Workflow illustrating the conjugation reaction between DBCO-RNA and 151UAA-GFP at varying molar ratios. (**D**) Gel shift analysis demonstrating the conjugation of DBCO-RNA with 151UAA-GFP at various molar ratios. (**E**) Quantitative analysis of the conjugation efficiency shown in (**D**). Statistical analysis was determined using one-way ANOVA followed by Dunnett's multiple comparisons test (n = 3, ns: not significant) (**F**) Workflow for evaluating the target-specific DNA-binding ability of RGG and DGG. T [ss]: target ssDNA. NT [ss]: non-target ssDNA. T [ds]: target dsDNA. (**G**) Gel shift analysis showing that RGG and DGG specifically bind to target ssDNA. (**H**) Quantitative analysis of DNA binding efficiency shown in (**G**). Statistical significance was determined using one-way ANOVA followed by Dunnett's multiple comparisons test (n = 3, ***P < 0.001).

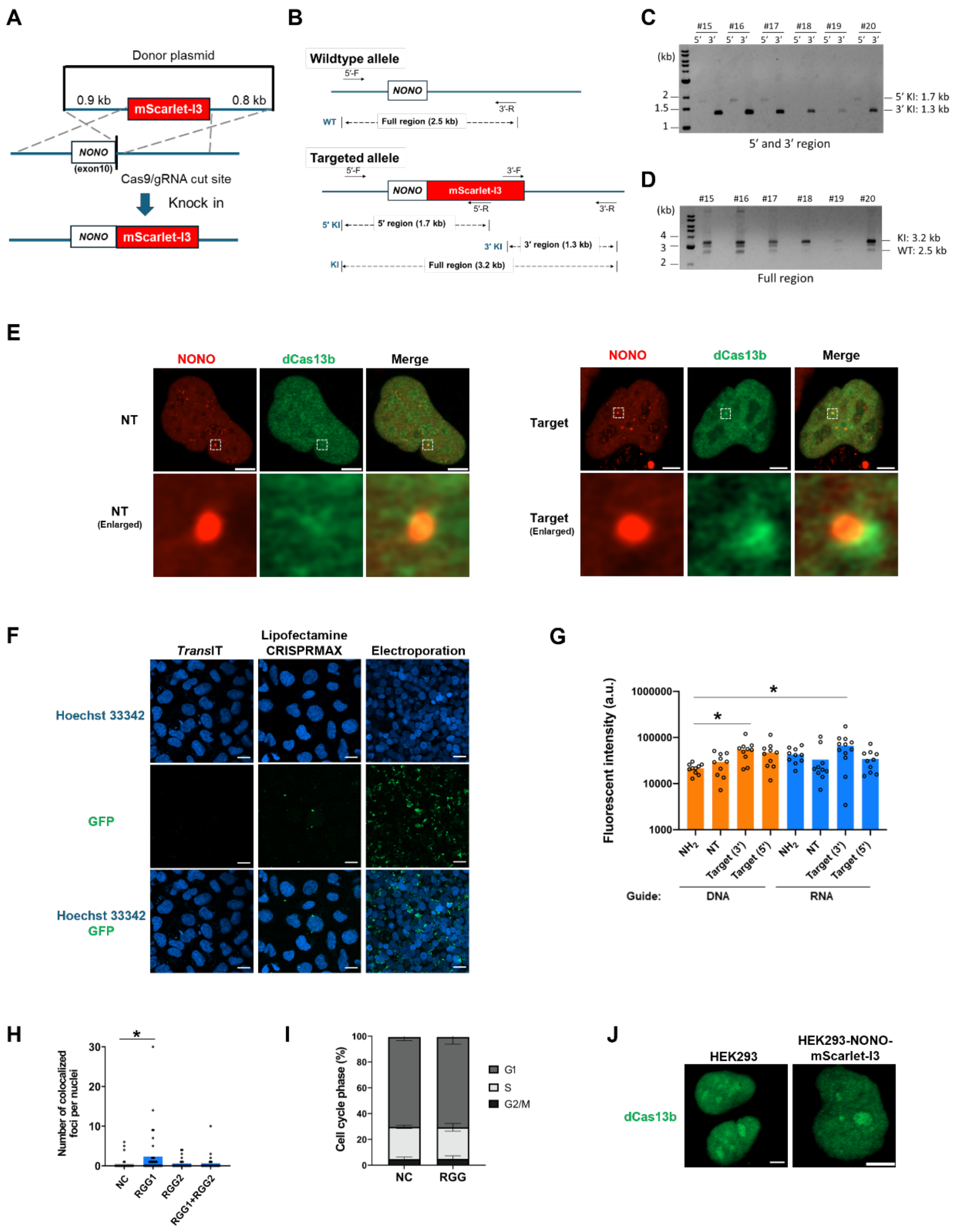

Figure S2: Generation of the HEK293-NONO-mScarlet-I3 cell line. (**A**) Schematic representation of donor construct, the target allele, and the resulting knock-in (KI) allele after successful integration. (**B**) Schematic diagram illustrating the verification strategy for the KI allele using genomic PCR. Black arrows indicate the positions of primers used for amplification. WT: wildtype. KI: knock-in. (**C**) Genome PCR analysis demonstrating amplification of the 5' and 3' flanking regions of the KI allele. Clone #20 was selected as

the reporter cell line. (**D**) Genome PCR analysis demonstrating successful amplification of the entire KI region. Clone #20 was identified as a heterozygous KI clone and used for the reporter cell line. (**E**) Representative fluorescence images showing dCas13b targeting *NEAT1* in live HEK293-NONO-mScarlet-I3 cells. *NEAT1* localization is visualized with NONO-mScarlet-I3 (red) and dCas13b (green), confirming specific labelling of *NEAT1*. NT: non-target. Scale bars: 5 µm. (**F**) Representative fluorescent images of HEK293 cells transfected with RGG using different transfection methods. Scale bars: 20 µm. (**G**) Quantitative analysis of green fluorescent intensity of NONO co-localization with DGG or RGG signals shown in **Figure 2D**. Statistical analysis was performed using the Kruskal–Wallis test followed by Dunn's multiple comparison test (NH_2-DNA: n = 10, NT-DNA: n = 10, Target-3'-DBCO-DNA: n = 10, Target-5'-DBCO-DNA: n = 10, NH_2-RNA: n = 10, NT-RNA: n = 10, Target-3'-DBCO-RNA: n = 12, Target-5'-DBCO-RNA: n = 10, $^*P < 0.05$). (**H**) Quantitative analysis of NONO co-localization with RGG. RGG1 uses Target-3'-DBCO-RNA, and RGG2 uses Target-3'-DBCO-RNA2. The total dose for each condition was 200 pmol (100 pmol of RGG1 and 100 pmol of RGG2). Statistical analysis was performed using the Kruskal–Wallis test followed by Dunn's multiple comparisons test (NC: n = 34, RGG1: n = 39, RGG2: n = 27, RGG1+RGG2: n = 29, $^*P < 0.05$). (**I**) Quantitative analysis of each cell cycle. Statistical analysis was performed using Student's t-test (n = 3). (**J**) Representative fluorescence images showing dCas13b (green) aggregates in live HEK293 and HEK293-NONO-mScarlet-I3 cells. Scale bars: 5 µm.

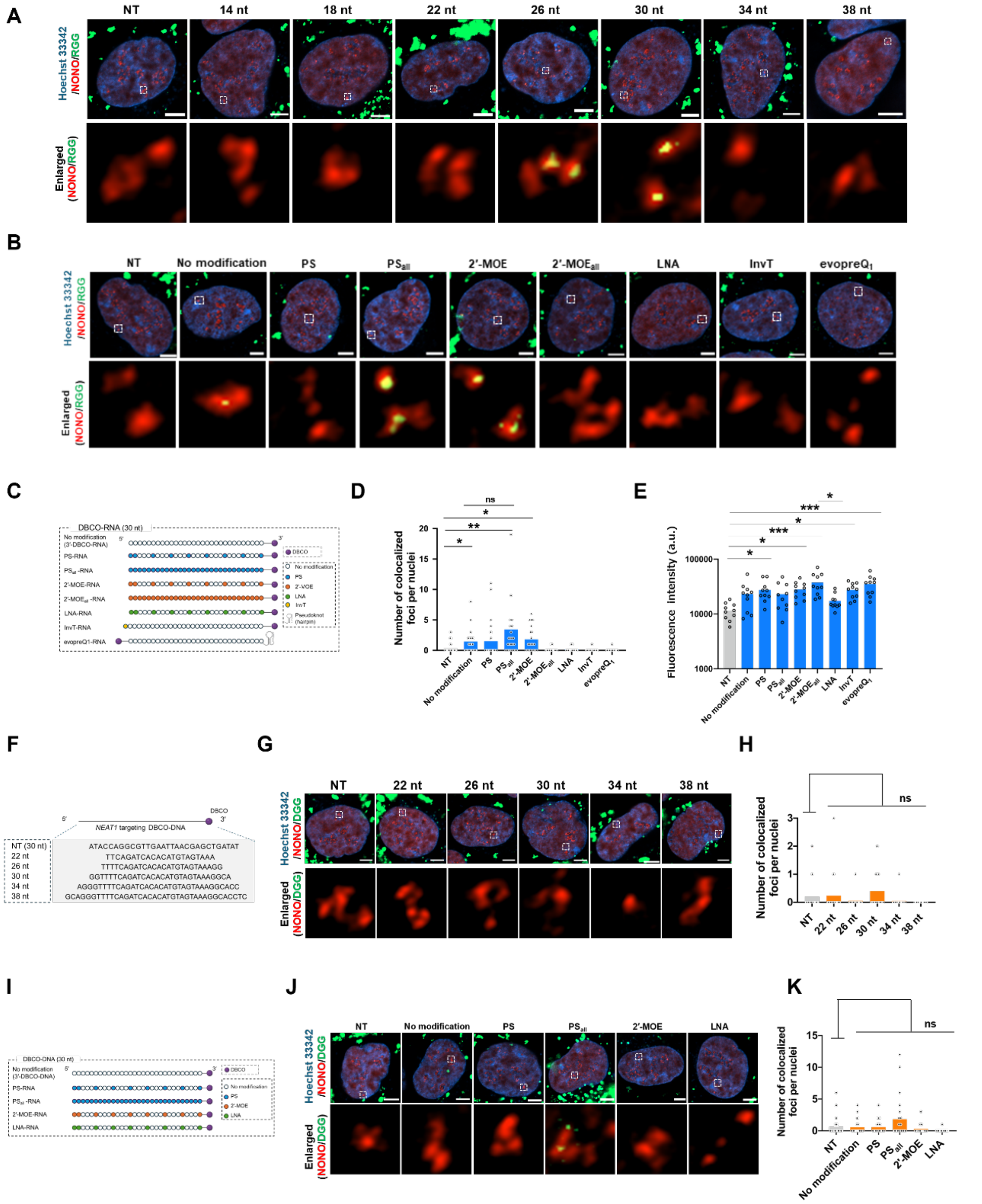

Figure S3: Optimization of *NEAT1* imaging with DGG. (**A**) Representative fluorescent images showing RGG conjugated with DBCO-RNA of varying lengths in live HEK293-NONO-mScarlet-I3 cells. NT: non-target. Scale bars: 5 µm. (**B**) Representative fluorescent images of live HEK293-NONO-mScarlet-I3 cells labelled with RGG conjugated to chemically modified DBCO-RNA. NT: non-target. Scale bars: 5 µm. (**C**) Schematic representation of additional chemical modifications introduced into DBCO-RNA, including phosphorothioate (PS), 2'-O-methoxyethyl (2'-MOE), locked nucleic acid (LNA),

and inverted thymidine (InvT) and evopreQ_1 (a stable pseudoknot-modified prequeosine$_1$-1 riboswitch aptamer). (**D**) Quantitative analysis of NONO co-localization with RGG conjugated to chemical modified DBCO-RNA, as shown in (**B**). Statistical analysis was performed using the Kruskal–Wallis test followed by Dunn's multiple comparisons test (NT: n =42, No modification: n = 39, PS: n = 25, PS_{all}: n = 25, 2'-MOE: n = 21, 2'-MOE_{all}: n = 39, LNA: n =25, InvT: n = 31, evopreQ_1: n = 30, *P < 0.05, **P < 0.01, ns: not significant). (**E**) Quantitative analysis of green fluorescence intensity using RNA imaging tools shown in (**B**). Statistical analysis was performed using the Kruskal–Wallis test followed by Dunn's multiple comparison test (NT: n =10, No modification: n = 10, PS: n = 10, PS_{all}: n = 10, 2'-MOE: n = 10, 2'-MOE_{all}: n = 10, LNA: n =10, InvT: n = 10, evopreQ_1: n = 10, *P < 0.05, ***P < 0.001). (**F**) Schematic illustration of DBCO-DNA length optimization for imaging performance. (**G**) Representative fluorescent images of DGG conjugated with DBCO-DNA of varying lengths in live HEK293-NONO-mScarlet-I3 cells. NT: non-target. Scale bars: 5 µm. (**H**) Quantitative analysis of NONO co-localization with DGG conjugated to DBCO-DNA of varying lengths, as shown in (**G**). Statistical analysis was performed using the Kruskal–Wallis test followed by Dunn's multiple comparisons test (NT: n = 20, 22 nt: n = 21, 26 nt: n = 18, 30 nt: n = 20, 34 nt: n = 20, 38 nt: n = 20, ns: not significant). (**I**) Schematic representation of additional chemical modifications introduced into DBCO-DNA for optimization, including phosphorothioate (PS), 2'-*O*-methoxyethyl (2'-MOE), and locked nucleic acid (LNA). (**J**) Representative fluorescence images of DGG conjugated to chemically modified DBCO-RNA in live HEK293-NONO-mScarlet-I3 cells. NT: non-target. Scale bars: 5 µm. (**K**) Quantitative analysis of NONO co-localization with DGG conjugated to chemically modified DBCO-DNA, as shown in (**J**). Statistical analysis was performed using the Kruskal–Wallis test followed by Dunn's multiple comparisons test (NT: n = 25, No modification: n = 25, PS: n = 24, PS_{all}: n = 24, 2′ -MOE: n = 19, LNA: n = 25, *P < 0.05).

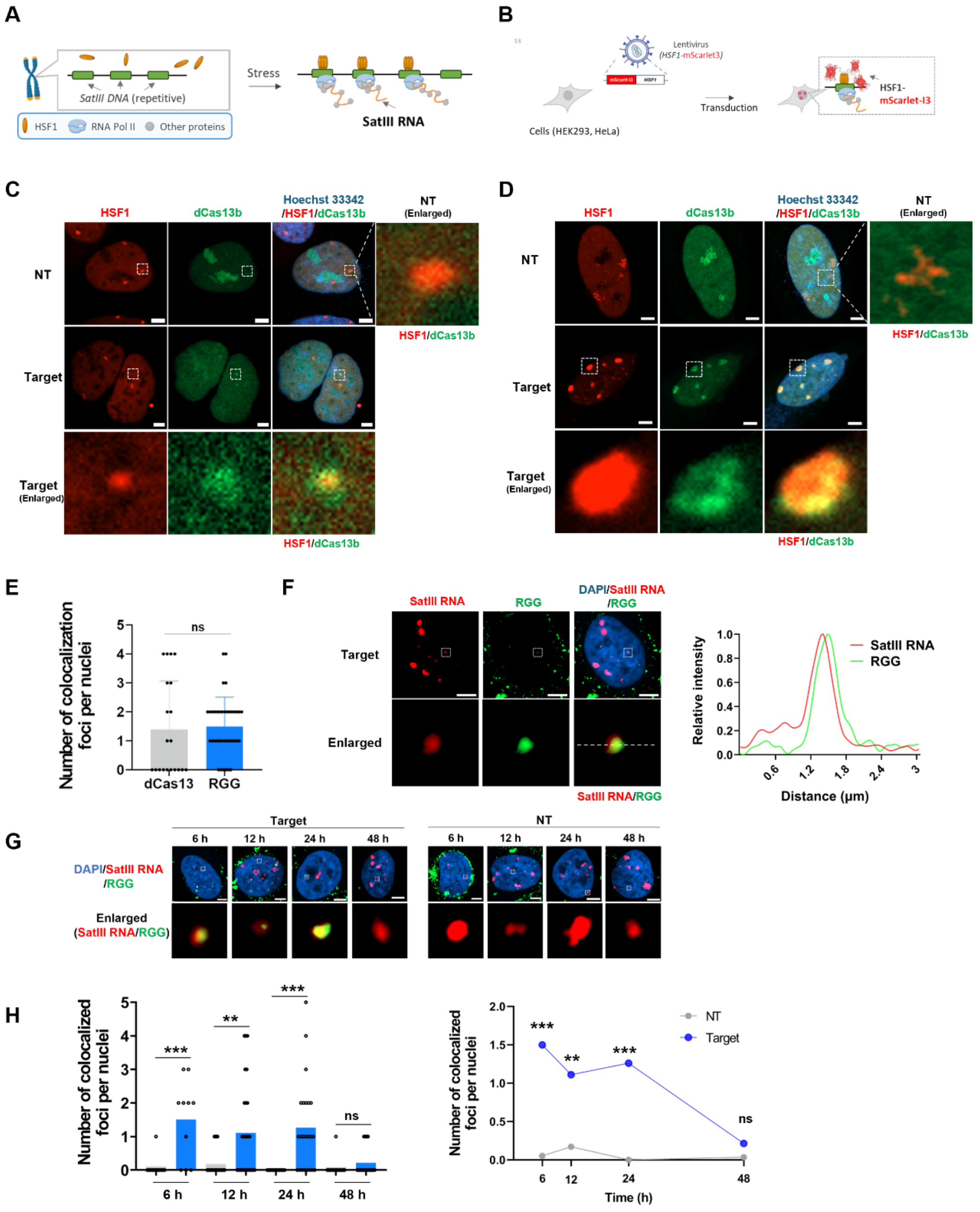

Figure S4: Visualization of SatIII RNA in living cells. (**A**) Schematic representation of SatIII RNA induction during cellular stress. Heat shock factor 1 (HSF1) forms a trimer, binds to the *SatIII DNA*, and activates SatIII RNA transcription by RNA Polymerase II (RNA Pol II). The transcribed RNA remains localized at the transcription site, even after transcription concludes. (**B**) Generation of a red fluorescent reporter cell for SatIII RNA detection. SatIII RNA is visualized in red. (**C**) Representative fluorescence images of SatIII RNA-targeted dCas13b introduced into live HEK293-HSF1-mScarlet-I3 cells following SA treatment (100

μM for 1 h). The top panel shows non-targeting dCas13b, and the bottom panel shows SatIII RNA-targeting dCas13b. NT: non-target. Scale bars: 5 μm. (**D**) Representative fluorescence images of SatIII RNA-targeted dCas13b introduced into live HeLa-HSF1-mScarlet-I3 cells after SA treatment (300 μM for 1 h). The top panel shows non-targeting dCas13b, and the bottom panel shows SatIII RNA-targeting dCas13b. NT: non-target. Scale bars: 5 μm. (**E**) Quantitative analysis of HSF1 co-localization with RNA imaging tools. Statistical analysis was performed using the Mann–Whitney test (dCas13: n = 20, RGG: n = 38, ns: not significant). (**F**) Representative fluorescence images of WT HeLa cells transfected with RGG and treated with SA (150 μM for 1 h). The line profiles to the right illustrate the red (SatIII RNA) and green (RGG) fluorescent signals, confirming their spatial co-localization. Scale bars: 5 μm. (**G**) Representative fluorescence images of co-localization between RGG and SatIII RNA-FISH foci over time, measured from electroporation to fixation. (**H**) Quantitative analysis of co-localization between RGG and SatIII RNA-FISH foci over time, measured from electroporation to fixation, as shown in (**G**) Left panel shows dot plots of co-localized foci counts at each time point, with bars indicating mean values. Right panel presents the mean co-localized foci counts connected by a line graph to illustrate the temporal trend. Statistical analysis was performed using the Mann–Whitney test (NT: gray — 6 h: n = 19, 12 h: n = 35, 24 h: n = 35, 48 h: n = 29; Target: blue — 6 h: n = 10, 12 h: n = 27, 24 h: n = 23, 48 h: n = 28, $^{**}P < 0.01$, $^{***}P < 0.001$, ns: not significant).

Supplementary Table S1. Codon-optimized 151TAG-GFP DNA sequence.

ATGAAAAGGACAGCTGATGGATCAGAATTTGAGTCCCCGAAAAAGAAGCGCAAAGTGGAGAGCGGTCTGCGTAGCGACAATATGGTATCAAAAGG
AGAAGAGCTATTTACAGGCGTAGTTCCGATTCTGGTGGAGCTGGATGGCGACGTGAACGGTCATAAATTCTCCGTGAGCGGTGAGGGCGAGGGTGA
CGCCACGTACGGCAAGTTGACCCTGAAGTTTATTTGTACCACCGGTAAGCTTCCGGTGCCGTGGCCTACGCTCGTTACTACGTTGACCTATGGTGTCC
AATGCTTTTCTCGTTATCCGGATCACATGAAACAACATGATTTCTTCAAGAGCGCAATGCCGGAAGGCTACGTGCAAGAACGTACAATTTTTTTCAAG
GACGATGGAAACTACAAGACCCGCGCAGAAGTTAAATTCGAGGGCGACACCCTGGTCAACCGCATTGAGCTGAAGGGTATCGACTTCAAAGAGGAT
GGCAACATCCTGGGCCACAAATTGGAATATAACTACAATAGCCATAACGTGTAGATTATGGCGGATAAACAGAAAAACGGCATCAAGGTGAATTTTA
AGATCCGTCATAATATCGAGGACGGTAGCGTCCAGCTGGCGGACCACTATCAGCAGAATACCCCGATCGGTGACGGTCCGGTTCTGTTGCCAGACAA
CCACTACCTGTCCACCCAGAGCGCTCTGTCGAAAGATCCGAATGAAAAACGTGACCACATGGTTCTGTTGGAATTTGTTACTGCGGCGGGTATCACCC
TGGGTATGGATGAATTATATAAATCAGGACTAAGGAGTGATAAAAGAACAGCTGACGGTAGCGAGTTCGAGTCCCCGAAAAAGAAACGTAAGGTGG
AACATCACCACCATCACCACTAA

Blue text indicates bpNLS, green indicates GFP, red indicates TAG codon and purple indicates 6xHIs.

Supplementary Table S2. The target sequence of CRISPR-Cas used in this study.

Target	Sequence (5′ to 3′)	Purpose
NONO	ACACTGCAACTTATTAGTATCGG	Cas9 mediated knock-in
NEAT1	GGTTTTCAGATCACACATGTAGTAAAGGCA	dCas13b mediated RNA imaging
SatIII RNA	GTTGAATCCATTCCATTGCATTCCATTCAT	dCas13b mediated RNA imaging
Non-Target	GCAGGGTTTTCCCAGTCACGACGTTGTAAA	dCas13b mediated RNA imaging

The underlined part is a PAM sequence of Cas9.

Supplementary Table S3. Primers used in this study.

Name	Sequence (5′ to 3′)	Purpose
NONO cDNA-LHR (F)	TATTAGAGACTGAGTTTGACCACGTT	Amplification of *NONO* cDNA
NONO cDNA-LHR (R)	GTATCGGCGACGTTTGTTTGG	Amplification of *NONO* cDNA
NONO cDNA-RHR (F)	TAATAAGTTGCAGTGTCTAGTTTCT	Amplification of *NONO* cDNA
NONO cDNA-RHR (R)	AAAGATGGAGGCAAGTTAACACAAG	Amplification of *NONO* cDNA
NONO gRNA (F)	TGGCTTTATATATCTTGTGGAAAGGACGAAACACCG CACTGCAACTTATTAGTAT	Construction of *NONO*-targeting sgRNA expression vector for Cas9
NONO gRNA (R)	GCCTTATTTTAACTTGCTATTTCTAGCTCTAAAACAT	Construction of *NONO*-targeting sgRNA expression

	ACTAATAAGTTGCAGTGC	vector for Cas9
NONO-mScarlt-I3 (LHR, F)	TAAAGGGACTAGTCCTGCAGGTATTAGAGACTGAGTTTGACCACGTTGGTCAGGCTGTTCT	Construction of NONO-mScarlet-I3 knock-in donor expression vector
NONO-mScarlt-I3 (LHR, R)	CTCGGTGCTATCCATGTATCGGCGACGTTTGTTTGGGGCAAATTCAGC	Construction of NONO-mScarlet-I3 knock-in donor expression vector
NONO-mScarlt-I3 (F)	ATGGATAGCACCGAGGCAGTGATCAAGGAGTTCA	Construction of NONO-mScarlet-I3 knock-in donor expression vector
NONO-mScarlt-I3 (R)	CACTGCAACTTATTAGGAGCCACCGGAGCCGCCG	Construction of NONO-mScarlet-I3 knock-in donor expression vector
NONO-mScarlt-I3 (RHR, F)	TAATAAGTTGCAGTGTCTAGTTTCTCAAAACCCTTAAAAGAAGG	Construction of NONO-mScarlet-I3 knock-in donor expression vector
NONO-mScarlt-I3 (RHR, R)	CGAATTGAATTTAGCGGCCGCAAAGATGGAGGCAAGTTAACACAAGATTTTTTTTAAAGATACACT	Construction of NONO-mScarlet-I3 knock-in donor expression vector
NONO-KI (5′-F)	ACCTGCCACTATGATGCCGGATGGAAC	Validation of knock-in
NONO-KI (5′-R)	CCCAGCCCATTGTCCTCTTCTGCATTACGG	Validation of knock-in
NONO-KI (3′-F)	GGACTACACCGTGGTGGAACAGTACGAACGCTC	Validation of knock-in
NONO-KI (3′-R)	CAACCCCTCCAGCCATTTGATGCACCT	Validation of knock-in
HSF1 cDNA (F)	TGTGAAGCCCCAACCAAACCACAGGACA	Amplification of *HSF1* cDNA
HSF1 cDNA (R)	CGCTCCCTCCGCCTATTCCCT	Amplification of *HSF1* cDNA
HSF1-mScarlt-I3 (N, F)	CAGCTGGCTACCGGTGCCACCATGGATAGCACCGAG	Construction of lentivirus vector for HSF1-mScarlet-I3 reporter cell
HSF1-mScarlt-I3 (N, R)	GGAGCCGCCGCCACCGGAGCCACCGGAGCCGCCG	Construction of lentivirus vector for HSF1-mScarlet-I3 reporter cell
HSF1-mScarlt-I3 (C, F)	GGTGGCGGCGGCTCCATGGATCTGCCCGTGGGCCCC	Construction of lentivirus vector for HSF1-mScarlet-I3 reporter cell
HSF1-mScarlt-I3 (C, R)	TCATTGGTCTTAAAGGTACCCTAGGAGACAGTGGGGTCCTTGG	Construction of lentivirus vector for HSF1-mScarlet-I3 reporter cell
NEAT1-gRNA (F)	CACCGGTTTTCAGATCACACATGTAGTAAAGGCA	Construction of *NEAT1*-targeting sgRNA expression vector for dCas13b
NEAT1-gRNA (R)	CAACTGCCTTTACTACATGTGTGATCTGAAAACC	Construction of *NEAT1*-targeting sgRNA expression vector for dCas13b
SatIII RNA-gRNA (F)	CACCGTTGAATCCATTCCATTGCATTCCATTCAT	Construction of SatIII RNA-targeting sgRNA

		expression vector for dCas13b
SatIII RNA -gRNA (R)	CAACATGAATGGAATGCAATGGAATGGATTCAAC	Construction of SatIII RNA-targeting sgRNA expression vector for dCas13b
NT-gRNA (F)	CACCGCAGGGTTTTCCCAGTCACGACGTTGTAAA	Construction of Non-targeting sgRNA expression vector for dCas13b
NT-gRNA (R)	CAACTTTACAACGTCGTGACTGGGAAAACCCTGC	Construction of Non-targeting sgRNA expression vector for dCas13b
GAPDH-F	AATCCCATCACCATCTTCCA	RIP-qPCR (Internal control)
GAPDH-R	TGGACTCCACGACGTACTCA	RIP-qPCR (Internal control)
SatIII RNA-F	TATGAATTCAATCAACCCGAGTGCAATCGAA	RIP-qPCR
SatIII RNA -R	TATGGATCCTTCCATTCCATTGCTGTACTCG	RIP-qPCR

Supplementary Table S4. Nucleic acids and chemically modified nucleic acids used in this study.

Name	Sequence (5′ to 3′)	Purpose
Target RNA [Tel26]	GCUAGAUCCGUAGGGUUAGGGUUAGGGUUAGGGUUAACCUCCGACC	ssRNA binding Assay
Non-Target RNA [Tel26]	GCUAGAUCCGGGUUGGAUGUAGAGUAUGGUAGUGGUACCUCCGACC	ssRNA binding Assay
Target DNA [Tel26]	GCTAGATCCGTAGGGTTAGGGTTAGGGTTAGGGTTAACCTCCGACC	DNA binding Assay
Target DNA [compDNA, Tel26]	GGTCGGAGGTTAACCCTAACCCTAACCCTAACCCTACGGATCTAGC	DNA binding Assay
Non-Target DNA [Tel26]	GCTAGATCCGGGTTGGATGTAGAGTATGGTAGTGGTACCTCCGACC	DNA binding Assay
Tel26-DBCO-RNA	UAACCCUAACCCUAACCCUAACCCUA-DBCO	ssRNA, DNA binding Assay
Tel26-NH_2-RNA	UAACCCUAACCCUAACCCUAACCCUA- NH_2	RGG, DGG formation Assay
Tel26-DBCO-DNA	TAACCCTAACCCTAACCCTAACCCTA-DBCO	ssRNA, DNA binding Assay
Tel26-NH_2-DNA	TAACCCTAACCCTAACCCTAACCCTA- NH_2	RGG, DGG formation Assay
Target-3′-DBCO-RNA	GGUUUUCAGAUCACACAUGUAGUAAAGGCA-DBCO	*NEAT1* imaging
Target-5′-DBCO-RNA	DBCO-GGUUUUCAGAUCACACAUGUAGUAAAGGCA	*NEAT1* imaging
NH_2-RNA	GGUUUUCAGAUCACACAUGUAGUAAAGGCA-NH_2	*NEAT1* imaging
NT-DBCO-RNA	AUACCAGGCGUUGAAUUAACGAGCUGAUAU-DBCO	Non-target control for *NEAT1*, SatIII RNA imaging
Target-3′-DBCO-DNA	GGTTTTCAGATCACACATGTAGTAAAGGCA-DBCO	*NEAT1* imaging

Target-5′-DBCO-DNA	DBCO-GGTTTTCAGATCACACATGTAGTAAAGGCA	*NEAT1* imaging
NH_2-DNA	GGTTTTCAGATCACACATGTAGTAAAGGCA-NH_2	*NEAT1* imaging
NT-DBCO-DNA	ATACCAGGCGTTGAATTAACGAGCTGATAT-DBCO	*NEAT1* imaging
Target-3′-DBCO-RNA2	AAUUGUUUGCAUCAUCCCCAAGUCAUUGGU-DBCO	*NEAT1* imaging
FITC-RNA	GGUUUUCAGAUCACACAUGUAGUAAAGGCAFITC	*NEAT1* imaging
NEAT1-DBCO14-RNA	GAUCACACAUGUAG-DBCO	*NEAT1* imaging
NEAT1-DBCO18-RNA	CAGAUCACACAUGUAGUA-DBCO	*NEAT1* imaging
NEAT1-DBCO22-RNA	UUCAGAUCACACAUGUAGUAAA-DBCO	*NEAT1* imaging
NEAT1-DBCO26-RNA	UUUUCAGAUCACACAUGUAGUAAAGG-DBCO	*NEAT1* imaging
NEAT1-DBCO34-RNA	AGGGUUUUCAGAUCACACAUGUAGUAAAGGCACC-DBCO	*NEAT1* imaging
NEAT1-DBCO38-RNA	GCAGGGUUUUCAGAUCACACAUGUAGUAAAGGCACCUC-DBCO	*NEAT1* imaging
NEAT1-DBCO22-DNA	TTCAGATCACACATGTAGTAAA-DBCO	*NEAT1* imaging
NEAT1-DBCO26-DNA	TTTTCAGATCACACATGTAGTAAAGG-DBCO	*NEAT1* imaging
NEAT1-DBCO34-DNA	AGGGTTTTCAGATCACACATGTAGTAAAGGCACC-DBCO	*NEAT1* imaging
NEAT1-DBCO38-DNA	GCAGGGTTTTCAGATCACACATGTAGTAAAGGCACCTC-DBCO	*NEAT1* imaging
PS-RNA	GGUUUUCAGAUCACACAUGUAGUAAAGGCA-DBCO	*NEAT1* imaging. Blue indicates phosphorothioate modifications
PS_{all}-RNA	GGUUUUCAGAUCACACAUGUAGUAAAGGCA-DBCO	*NEAT1* imaging. Blue indicates phosphorothioate modifications
2′-MOE-RNA	GGUUUUCAGAUCACACAUGUAGUAAAGGCA-DBCO	*NEAT1* imaging. Red indicates 2′′-O-methoxyethyl modifications
2′-MOE_{all}-RNA	GGUUUUCAGAUCACACAUGUAGUAAAGGCA-DBCO	*NEAT1* imaging. Red indicates 2′-O-methoxyethyl modifications
LNA-RNA	GGUUUUCAGAUCACACAUGUAGUAAAGGCA-DBCO	*NEAT1* imaging. Green indicates locked nucleic acids modifications
InvT-RNA	InvT-GGUUUUCAGAUCACACAUGUAGUAAAGGCA-DBCO	*NEAT1* imaging.
$evopreQ_1$-RNA	DBCO-GGUUUUCAGAUCACACAUGUAGUAAAGGCAAUAAAGAGCGCGGUUCUAUCUAGUUACGCGUUAAACCAACUAGAA	*NEAT1* imaging. Purple indicates $evopreQ_1$/
PS-DNA	GGTTTTCAGATCACACATGTAGTAAAGGCA-DBCO	*NEAT1* imaging. Blue indicates

		phosphorothioate modifications
PS_{all}-DNA	GGTTTTCAGATCACACATGTAGTAAAGGCA-DBCO	*NEAT1* imaging. Blue indicates phosphorothioate modifications
2′-MOE-DNA	GGTTTTCAGATCACACATGTAGTAAAGGCA-DBCO	*NEAT1* imaging. Red indicates 2′-O-methoxyethyl modifications
LNA-DNA	GGTTTTCAGATCACACATGTAGTAAAGGCA-DBCO	*NEAT1* imaging. Green is locked nucleic acids modifications
SatIII RNA-DBCO-RNA	GUUGAAUCCAUUCCAUUGCAUUCCAUUCAU-DBCO	SatIII RNA imaging
Cy3-SatIII-1	GTTGAATCCATTCCATTGCATTCCATTCAT-Cy3	SatIII RNA FISH
Cy3-SatIII-2	GATTCCAATCCATGCCATTCCAC-Cy3	SatIII RNA FISH
